# Quantitation Overcoming Matrix Effects of Lipophilic Toxins in *Mytilus galloprovincialis* by Liquid Chromatography-Full Scan High Resolution Mass Spectrometry Analysis (LC-HR-MS)

**DOI:** 10.3390/md20020143

**Published:** 2022-02-15

**Authors:** Camila Q. V. Costa, Inês I. Afonso, Sandra Lage, Pedro Reis Costa, Adelino V. M. Canário, José P. Da Silva

**Affiliations:** 1 Centre of Marine Sciences (CCMAR/CIMAR LA), University of Algarve, Campus de Gambelas, 8005-139 Faro, Portugal; camilaqvdacosta@gmail.com (C.Q.V.C.); inesiafonso13@gmail.com (I.I.A.); smlage@ualg.pt (S.L.); prcosta@ipma.pt (P.R.C.); acanario@ualg.pt (A.V.M.C.); 2 Portuguese Institute for the Sea and Atmosphere (IPMA), Av. Brasília, 1449-006 Lisbon, Portugal

**Keywords:** liquid chromatography-mass spectrometry (LC-MS), liquid chromatography-high resolution mass spectrometry (LC-HR-MS), full scan, matrix effects, okadaic acid (OA), dinophysistoxin-1 (DTX-1), dinophysistoxin-2 (DTX-2), pectenotoxin-2 (PTX-2), azaspiracid-1 (AZA-1), yessotoxin (YTX)

## Abstract

The analysis of marine lipophilic toxins in shellfish products still represents a challenging task due to the complexity and diversity of the sample matrix. Liquid chromatography coupled with mass spectrometry (LC-MS) is the technique of choice for accurate quantitative measurements in complex samples. By combining unambiguous identification with the high selectivity of tandem MS, it provides the required high sensitivity and specificity. However, LC-MS is prone to matrix effects (ME) that need to be evaluated during the development and validation of methods. Furthermore, the large sample-to-sample variability, even between samples of the same species and geographic origin, needs a procedure to evaluate and control ME continuously. Here, we analyzed the toxins okadaic acid (OA), dinophysistoxins (DTX-1 and DTX-2), pectenotoxin (PTX-2), yessotoxin (YTX) and azaspiracid-1 (AZA-1). Samples were mussels (*Mytilus galloprovincialis*), both fresh and processed, and a toxin-free mussel reference material. We developed an accurate mass-extracted ion chromatogram (AM-XIC) based quantitation method using an Orbitrap instrument, evaluated the ME for different types and extracts of mussel samples, characterized the main compounds co-eluting with the targeted molecules and quantified toxins in samples by following a standard addition method (SAM). An AM-XIC based quantitation of lipophilic toxins in mussel samples using high resolution and accuracy full scan profiles (LC-HR-MS) is a good alternative to multi reaction monitoring (MRM) for instruments with HR capabilities. ME depend on the starting sample matrix and the sample preparation. ME are particularly strong for OA and related toxins, showing values below 50% for fresh mussel samples. Results for other toxins (AZA-1, YTX and PTX-2) are between 75% and 110%. ME in unknown matrices can be evaluated by comparing their full scan LC-HR-MS profiles with those of known samples with known ME. ME can be corrected by following SAM with AM-XIC quantitation if necessary.

## 1. Introduction

Marine biotoxins are naturally occurring compounds mostly produced by microalgae—namely, diatoms and dinoflagellates, which accumulate in bivalves as a result of their filtering-feeding activity [1,2]. The consumption of contaminated bivalves, such as mussels, clams or oysters, can result in severe illness. One of the most common is diarrhetic shellfish poisoning (DSP) [3]. DSP toxins are lipophilic compounds and can be grouped into okadaic acid (OA) and its derivatives (dinophysistoxins, DTXs), polyether-lactones of the pectenotoxin group (PTXs), and sulphated cyclic polyether brevetoxin-like compounds, known as yessotoxins (YTXs). Another group of lipophilic toxins are the azaspiracids (AZA group), which lead to azaspiracid poisoning (AZP) and show symptoms similar to those induced by DSP toxins [4,5]. Lipophilic toxins of OA, YTX and AZA groups are currently regulated by the European Union (EU) by setting their maximum levels in marketed shellfish products [6,7]. An EU Standard Operation Procedure is available for the analysis of these compounds, which involves the extraction with methanol followed by liquid chromatography coupled with electrospray tandem mass spectrometry (LC-ESI-MS/MS) analysis [8]. A step involving alkaline hydrolysis is necessary to quantify esterified OA, DTX-1 and DTX-2. 

Liquid chromatography coupled with mass spectrometry (LC-MS)is an important tool for the identification and quantitation of analytes, such as biotoxins, in complex mixtures [4,9]. The high sensitivity and specificity, which ensures unambiguous identification and quantification, together with the high selectivity of tandem MS, has led to high-throughput analysis incorporating little or no sample preparation. However, the high selectivity of liquid chromatography coupled with tandem mass spectrometry (LC-MS/MS) does not guarantee an effective elimination of interferences from endogenous compounds of samples, such as bivalves samples. Quantitative analysis using electrospray ionization (ESI), or atmospheric pressure chemical ionization (APCI) can be substantially affected due to signal suppression or enhancement caused by matrix compounds. The phenomenon is known as “matrix effects” (ME) and can lead to erroneous quantitation [10,11].

Matrix effects are attributed to organic and/or inorganic compounds of sample co-eluting with the analytes, affecting their ionization efficiency and therefore the overall system response [10]. Both endogenous materials present in samples after extraction and compounds present in the LC mobile phase can be responsible for this behaviour. ME depend on the sample characteristics, sample preparation, chromatographic separation and ionization conditions, and therefore require evaluation for each matrix, sample preparation method and analytical conditions. 

ME can be evaluated by infusing a solution of the analyte at a point after the chromatographic column during the injection of a sample [10,12]. The monitorization of the instrument response during a sample run allows the identification of time windows where the analyte signal is enhanced or suppressed. The gathered profile is usually compared to one obtained after injection of a blank. However, the most common procedure to evaluate ME is to compare the signal of the target compound spiked in an analyte-free sample extract with the response obtained from a standard solution at the same concentration [5,12]. Differences in the responses indicate ion suppression or enhancement. ME can be quantified using the equation:

(1) $$\mathrm{ME}\left(\%\right)=\frac{B}{A}\times 100$$

where *A* is the average peak area of the standard solution and *B* represents the average peak area in the spiked extract. If ME is equal to 100%, no matrix effect is present. If ME is higher than 100% enhancement effects are present while ME lower than 100% means ion suppression is taking place.

Several procedures have been suggested to decrease the interferences of co-eluting matrix compounds. Sample cleanup of potential interference compounds is a logical strategy [13,14,15,16]. However, this procedure can be time-consuming, particularly for complex extracts possessing compounds with different properties. Alternatively, the chromatographic conditions can be modified to remove the co-eluting matrix compounds from the time window of the analytes [14]. Finally, instrumental modification such as the change of the ion source type can also decrease ME [10]. Either way, when ME are present several approaches should be carried out to correctly quantitate the target compounds. The most efficient procedure to compensate for ME in LC-MS or LC-MS/MS is the isotope dilution method, which involves the addition of stable isotope-labeled analytes as internal standards [17]. However, isotope-labeled standards are available for a few compounds and are expensive. Alternatively, ME can be corrected by using a SAM. In this case, sample extracts are spiked with known amounts of targeted compounds and matrix modified calibration curves are generated [5,18]. However, this procedure requires longer processing and analysis times. 

Several studies have been reporting ME during LC-MS analysis of lipophilic marine biotoxins in bivalves [5,13,14,15,18,19]. Most studies propose strategies to decrease ME, but their removal is never complete and corrections to ME are always necessary to achieve accurate results. Little attention has been given to the identity and reactivity of compounds responsible for the ME. The identity and reactivity of co-eluting compounds are key information in understanding, and therefore predicting and controlling ME. 

**Scheme 1:**
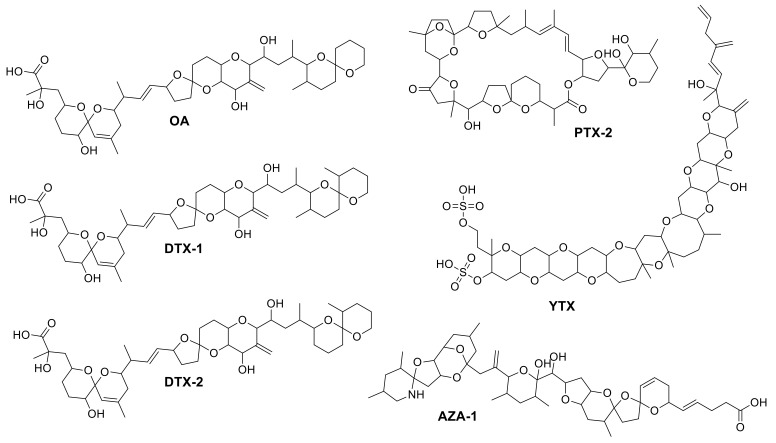
Structures of OA, DTX-1, DTX-2, PTX-2, YTX and AZA-1.

In this work, we used the full scan liquid chromatography coupled with high resolution mass spectrometry (LC-HR-MS) profiles to quantify toxins in mussel extracts and to evaluate ME. The sample properties and preparation—namely, the size of organisms and processing procedure—on the identity and transformation of compounds co-eluting with lipophilic toxins during LC-MS analysis of mussel extracts were studied. The chosen toxins were OA, DTX-1, DTX-2, YTX and AZA-1 and the recently deregulated PTX-2 (Scheme 1) [7]. The ME were corrected by following a SAM. 

## 2. Results

### 2.1. Accurate Mass-Extracted Ion Chromatogram (AM-XIC) Based Quantitation

**Figure 1:**
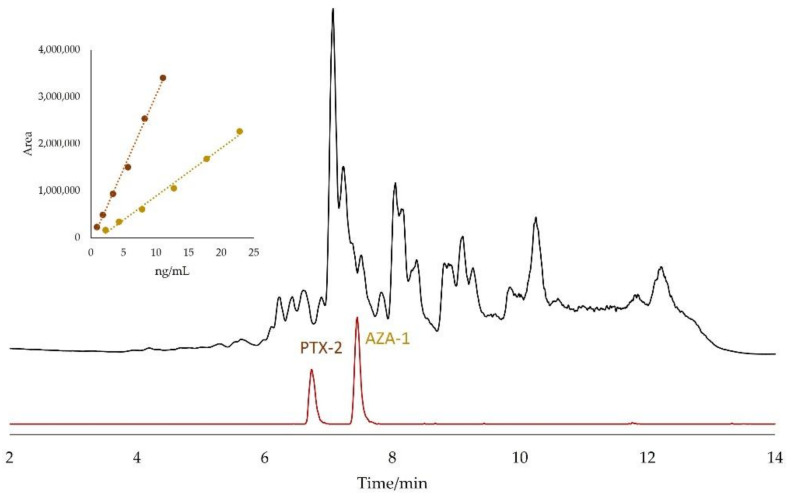
LC-HR-MS full scan positive profile of a non-hydrolyzed mussel methanolic extract (black) and the correspondent XIC (red) taking the *m/z* values 842.5049 and 876.5104 with ±5 ppm window. Concentrations of AZA-1 and PTX-2 were 1.45 ng/mL and 4.33 ng/mL, respectively. The insert shows the calibration curves for both toxins in this sample.

Figure 1 shows a typical profile obtained under full scan positive LC-HR-MS of a non-hydrolyzed sample extract of fresh mussels. The single-ion chromatogram extracted using the *m/z* values expected for AZA-1 and PTX-2, 842.5049 and 876.5104, respectively, is also presented. For comparison, the full scan profile as well as the XIC obtained for a hydrolyzed extract are presented in Figure S1 (Supplementary Materials, SM). The AM-XIC revelated signals at 6.75 min and 7.45 min, assigned to PTX-2 and AZA-1, respectively. The absence of other signals confirms the selectivity of the procedure. As expected, the full scan profile of the methanolic solution of standards showed an absence of strong MS signals (Figure S1a, SM). However, the full scan profile of the hydrolyzed extract (Figure S1b, SM) was significantly different from the non-hydrolyzed full scan profile, indicating that the hydrolysis process strongly modifies the matrix composition. The insert of Figure 1 contains the calibration curves, showing a linear response for AZA-1 and PTX-2 obtained after their addition to this matrix. 

For comparison purposes, an MRM method using the instrument linear trap (ITMS) was also developed for all toxins. The choice of MRM transitions was based on the collision-induced dissociation (CID) spectra obtained by infusion of the pure standards of individual biotoxins (Figures S2–S7 and Table S1, SM). Limits of quantification (LOQs) are similar for both AM-XIC and MRM methods (Table S1, SM) and to values reported in the literature [20]. The lowest concentration of standards was not used for quantitation. The AM-XIC based quantitation was adopted in this work, as it allows for quantitation of the targeted molecules and the detection of compounds of the matrix potentially responsible for ME effects. 

### 2.2. Matrix Effects

**Figure 2:**
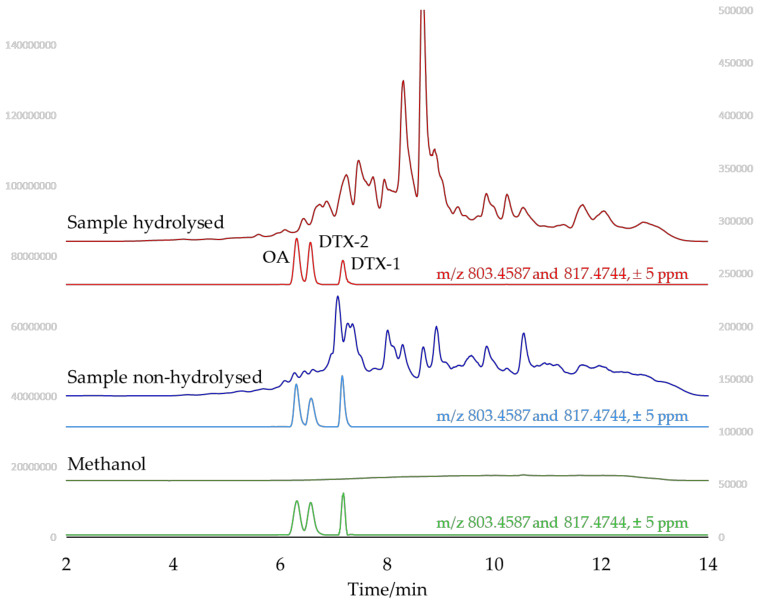
LC-HR-MS full scan negative profiles and correspondent XIC taking *m/z* 803.4587 and 817.4744 with ±5 ppm of a methanol standard (green) containing OA, DTX-1 and DTX-2, and a mussel extract before (blue) and after (red) alkaline hydrolysis, both fortified with the same compounds. The signal of YTX can obtained by following the same procedure (*m/z* 1141.4717, ±5 ppm) and was observed at 6.5 min (not shown). The concentration of OA was 3.77 ng/mL while it was 3.55 ng/mL for DTX-1 and DTX-2.

The compounds potentially responsible for ME were studied by full scan LC-HR-MS under negative and positive polarity. Figure 2 shows the profiles of a fresh mussel methanolic extract, before and after alkaline hydrolysis, under negative polarity. For comparison purposes, the profile of a standard solution in methanol is also included. AM-XIC of correspondent full scan traces are also shown. 

As observed for full scan profiles obtained under positive polarity (Figure 1 and Figure S1 (SM)), the full scan profile obtained in methanol had very low intensity signals and the full scan profiles before and after hydrolysis were significantly different, suggesting different ME for the different matrices. AM-XIC at *m/z* 803.4587 (OA and DTX-2) and 817.4744 (DTX-1) had clean signals at 6.31, 6.57 and 7.18 min, which were assigned to OA, DTX-2 and DTX-1, respectively. Comparison of the relative intensity of these signals indicated ME effects. For example, the hydrolyzed sample showed a signal of DTX-1 lower than that of OA while in the non-hydrolyzed sample the signal of DTX-1 was slightly higher than that of OA. 

Quantitative results of the matrix effect before and after alkaline hydrolysis are shown in Table 1. For comparison purposes, the same study was conducted with a reference material without toxins (CRM-Zero-Mus). Results are also presented in Table 1.

The highest ME under negative polarity was observed for OA and related toxins. DTX-1 showed a further ME increase in the hydrolyzed extract, consistent with the profiles shown in Figure 2. 

Results of ME for all analyzed fresh samples are presented in Table 2. The ME on DTX-1 is significantly higher in the hydrolyzed than in the non-hydrolized fresh mussel extracts.

**Table 1:** ME on toxins in hydrolyzed and non-hydrolyzed methanolic extracts of fresh mussels (*n* = 3).

Sample and Toxin	Non-Hydrolyzed Extract	% RSD (in-Batch)	Hydrolyzed Extract	% RSD (in-Batch)
Fresh mussels				
OA	45.2	2.4	41.1	1.9
DTX-1	41.9	3.9	20.5	4.2
DTX-2	43.8	3.3	37.5	2.5
YTX	108.6	7.6	100.1	10.1
AZA-1	80.6	4.5	76.6	8.2
PTX-2	96.7	5.5	98.1	8.8
Reference sample of mussels				
OA	51.5	0.9	33.7	5.9
DTX-1	35.5	0.7	25.6	4.3
DTX-2	50.5	4.6	26.4	8.0
YTX	57.6	9.5	76.0	4.0
AZA-1	71.0	1.5	71.2	4.4
PTX-2	65.5	0.8	77.2	3.3

RSD: Relative Standard Deviation.

**Table 2:** ME on toxins in hydrolyzed and non-hydrolyzed methanolic extracts of different mussel samples (*n* = 6) collect between September and December 2021 at Sagres area, Portugal.

Toxin	Non-Hydrolyzed Extract	% RSD (between Samples)	Hydrolyzed Extract	% RSD (between Samples)
OA	37 a	33	33 a	23
DTX-1	41 a	21	18 b	24
DTX-2	40 a	24	37 a	27
YTX	108 a	17	96 a	17
AZA-1	88 a	21	84 a	17
PTX-2	93 a	23	95 a	7

In the same line, values followed by the same letter do not differ significantly (*p* ≥ 0.05) according to ANOVA. RSD: Relative Standard Deviation.

### 2.3. Identification of Matrix Compounds

LC-HR-MS profiles of samples were acquired under data-dependent conditions in the positive and negative polarities. An extract of a fresh mussel sample, before and after hydrolysis was studied. More than 200 compounds were detected between 6.1 and 6.7 min, the retention time window of OA, DTX-2 and YTX. Figures S8 and S9 (SM) show average spectra in this time range, under negative and positive polarity, respectively. The main compounds tentatively identified in the non-hydrolyzed extracts were mostly lysophospholipids and included LPC 14:0, LPC-O 14:0, LPC 16:1, LPG 16:0, LPE 16:0 and oxygenated and hydroxylated docosahexaenoic acid, among others. As expected, alkaline hydrolysis strongly modified the spectra in this region (Figures S10 and S11, SM). Compounds showing a concentration increase upon hydrolysis in this time window included hydroxylated fatty acids. 

DTX-2 and AZA-1 are retained between 7.1 and 7.6 min. Other lysophospholipids such as LPC 18:1, LPC 17:0 LPC 18:1 and LPC-O 16:0 were tentatively identified in this time range (Figures S12 and S13, SM). Alkaline hydrolysis also changed the profiles in this time range (Figures S14 and S15, SM). These compounds appeared as very broad peaks, extending over 1 min, and in some cases 2 min. 

The gathered profiles and spectra were compared with those obtained for a toxin-free reference material (CRM-Zero-Mus). A full scan LC-HR-MS profile obtained under negative polarity is presented in Figure S16 (SM). CRM and fresh mussels sample show different profiles between 6.1 and 6.7 min and between 7.1 and 7.6 min indicating compounds co-eluting with the targeted molecules were also different. For example, in the range between 6.1 and 6.7 min the fresh samples showed a strong signal at *m/z* 359 that was not seen in the CRM (compare Figures S8 and S17).

### 2.4. Quantitation by the Standard Addition Method

To quantify OA and DTX-2 on pasteurized and thermally processed mussel samples of three different sizes (Mincha, Medium and Big) contaminated with these toxins, full scan LC-HR-MS profiles were obtained first (Figure S18, SM). The correspondent negative polarity spectra between 6.1 and 6.7 min, the retention time window of both toxins, are presented in Figure S19 (SM). Many signals were common to spectra present in all samples, but their relative intensities were generally different. The spectra of processed samples in this time window differed significantly from the one obtained for non-hydrolyzed and hydrolyzed fresh mussel samples, (Figures S8–S11 and S19) suggesting different ME. 

We further analyzed the co-eluting compounds to decide whether SAM was necessary to quantify OA and DTX-2 in contaminated samples. Detailed analysis of fresh and hydrolyzed samples spectra between 6.2 and 6.4 min (OA, Figures S20 and S21) and between 6.5 and 6.7 min (DTX-2, Figures S26 and S27) showed a group of signals between *m/z* 400 and 550 and another group with *m/z* below 400. The relative intensity of this latter group increased with hydrolysis and should be a result of hydrolysis and oxidation of fatty acids. These compounds are expected to compete with OA and DTX-2 for ionization under negative polarity. 

The spectra of the processed samples (Big, Medium, Mincha and Pasteurized) were significantly different (see Figures S22–S25 and S28–S31). Some signals were common to non-processed samples, but the compounds showing *m/z* below 400 were relatively less intense. The lower content of these compounds might cause ME lower than observed for non-processed samples (Table 1). This was confirmed after determination of ME for all processed samples (Table S2). ME values were higher than the observed for non-processed samples and in some cases were above 100%. Therefore, the use of ME correction obtained for non-processed samples will overestimate the contents of OA and DTX-2 in the processed contaminated ones.

**Figure 3:**
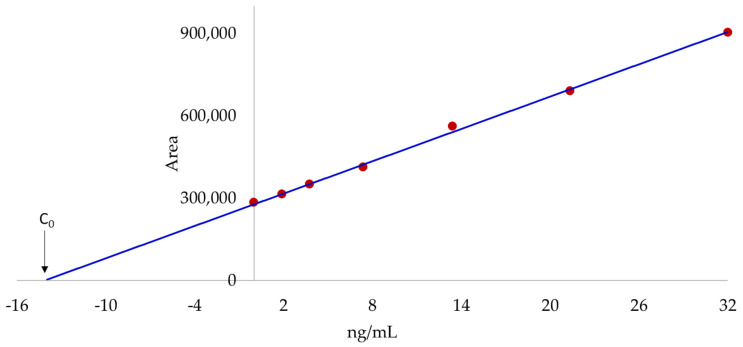
Area of OA as a function of the concentration in a sample of big mussels and fitting of data to a linear model (y = mx + b). m = 19,625; b = 277,546; *R*^2^ = 0.998; C_0_ = 14.1 ng/mL. Data were obtained from full scan profiles by following an AM-XIC procedure.

All contaminated samples were therefore analyzed by following a SAM to correct ME. In SAM the extracts of samples are spiked with known concentrations of toxins and calibration curves are obtained for each matrix. The analyte concentration (C_0_) can be obtained at the x-axis intercept of the linear regression (Figure 3) [21]. Spiking is, therefore, necessary for each sample matrix type. Table 3 shows the concentrations of OA and DTX-2 in the four mussel samples by following SAM. 

## 3. Discussion

The LC-MS quantitation based on the AM-XIC has been widely used [9,22,23]. As full scan LC-HR-MS are obtained, the procedure acquires information about the whole sample composition. This way, full scan profiles can be used for quantitation and identification of other toxins or compounds in the sample. AM-XIC showed a performance similar to the MRM method, indicating quantification can be readily achieved while keeping information from other compounds in the sample. 

ME were evaluated from the full scan profiles acquired to quantify the targeted toxins. Full scan profiles are strongly modified by the hydrolysis step used to quantify OA and related toxins. Evaluation of non-hydrolyzed and hydrolyzed extracts revealed that the ME are significantly different for DTX-1 toxin. Thus, an accurate measurement of this toxin requires the ME to be measured in the extract after hydrolysis. 

As expected, the identified molecules were mainly phospholipids, fatty acids and related compounds. Their distribution and intensity were strongly modified by the alkaline hydrolysis step. This was expected, as these compounds undergo thermal hydrolysis with subsequent release of fatty acids [24]. Furthermore, oxidation is also expected to take place, originating oxidation products as hydroxy unsaturated fatty acids. Lipids are known to cause ME [25], particularly long chain molecules, which is consistent with the observed ME, particularly in the negative polarity. However, the hydrolysis of extracts showed a strong decrease of some lipid classes, namely the lysophospholipids, while the ME remained constant for most toxins. These results suggest the hydrolysis products should also originate ME. 

Quantitation by LC-MS needs ME to be evaluated and corrected. As ME are related to compounds co-eluting with the analytes, knowledge of the main components of the matrix is important to evaluate and predict them. However, the composition of samples of living organisms depends on multiple parameters, such as the organism environment, age, species, life cycle and processing. Analytical information of each sample is necessary to perform this evaluation. 

The identification of compounds potentially responsible for ME in fresh and processed samples (Big, Medium, Mincha and Pasteurized), together with their spectral distributions and intensities indicated that determination of ME was necessary for processed samples. The decrease of ME in these samples was assigned to the lower relative contents of compounds possessing *m/z* values lower than 400 co-eluting at retention times of OA and DTX-2. A possible explanation for the lower content of these compounds and thus lower ME of processed mussel kernels (Big, Medium and Mincha), might be related to the processing procedure (see Section 4.2 below). Since the kernels are submitted to thermal processing and are then washed, compounds with *m/z* below 400 released during the thermal treatment will be removed, hence lowering the ME. Washing effect is also expected for the Pasteurized sample as water is released during the process. 

In conclusion, to evaluate ME and quantify toxins at the same time we propose sample analysis under LC-HR-MS full scan. Quantitation can be achieved by AM-XIC and ME can be evaluated from the comparison of the full scan spectra of the target sample and of the sample with known ME. The ME correction for a given matrix can be performed by following a SAM method. This procedure requires longer sample preparation and analysis time, as a calibration curve is necessary for each sample. However, it provides accurate results by correcting ME for each sample. Modern autosamplers can be programmed to prepare solutions saving preparation time. 

## 4. Materials and Methods

### 4.1. Materials

Water, acetonitrile and methanol (LC-MS grade) were from Carlo Erba, (Milan, Italy). Formic acid, ammonium formate, stearic acid, palmitic acid and lysophosphatidylcholines were from Sigma-Aldrich, (Darmstadt, Germany). OA, DTX-1, DTX-2, YTX and AZA-1certified reference materials (CRM) were purchased from CIFGA Laboratories, (Lugo, Spain). PTX-2 CRM, and mussel tissue reference material without toxins (CRM-Zero-Mus) were purchased from National Research Council (Halifax, NS, Canada).

### 4.2. Mussel Samples 

Fresh mussels, pasteurized mussels and mussel kernels (Big, Medium and Mincha, see below) were provided by Finisterra S.A. Six fresh mussel samples, collected between September and December 2021 in the area of Sagres, Portugal, were analyzed. Samples of mussels pasteurized with the shell and thermal treated mussel kernels containing OA and DTX-2 were also analyzed. The pasteurization process was the following: after washing, the mussels were placed in plastic bags at low pressure (500 mbar) and sealed. The packed mussels were then submitted to a 100 °C treatment under water vapor (650 mbar) in an autoclave. The temperature rose from 20 °C to 100 °C in 20 min, stayed at 100 °C for 7 min and then was allowed to cool to 20 °C inside the autoclave. A temperature above 90 °C for over than 90 s is ensured during the process. The thermal treatment of mussel kernels was performed in the following way: mussels with shells were first submitted to a cooking process in autoclave and water vapor (3.8 bar) at 147 °C for 48 s. This process opens the shells. Mussels with the shells were then placed in a 25% NaCl aqueous solution containing 10 ppm of hydrogen peroxide at a temperature between 28 and 32 °C. The kernel is released, washed to remove excess salt and calibrated according to the following sizes: Big—80 to 120 mussels per kg; Medium—120 to 160 mussels per kg and Mincha—>300 mussels per kg. 

### 4.3. Extraction and Addition of Standards

The extraction of toxins was performed by following the EU-Harmonised Standard Operating Procedure [8]. Briefly, 2 g of homogenized mussel tissue were extracted with methanol and filtered. To determine the total concentration of OA and DTX-2 the extracts were submitted to alkaline hydrolysis with NaOH at 76 °C for 40 min. After reaction, the extract was neutralized with acid and filtered. 

A working solution containing approximately 80 ng/mL of AZA-1; 200 ng/mL of OA, DTX-1, DTX-2 and PTX-2; and 500 ng/mL of YTX was prepared. The different matrices were then spiked with 2, 4, 8, 15, 25 and 40 μL of the working solution per 200 μL of matrix. The final concentration of targeted compounds was recalculated according to dilution. Spiked matrices were methanol, extracts of fresh mussels before and after the hydrolysis step, extracts of CRM-Zero-Mus, non-hydrolyzed and hydrolyzed, pasteurized mussels with shells and thermally processed mussel kernels with sizes Big, Medium and Mincha. Three replicates were analyzed for each sample batch. 

### 4.4. LC-MS Conditions

Extracts of samples were analyzed by LC-HR-MS. The chromatographic separation was performed on a Thermo Scientific ultimate 3000 UHPLC with a Waters Acquity Premier BEH C18 column (2.1 × 50 mm, 1.7 mm) and using a water (A)/acetonitrile (B) mobile phase containing 0.05% of formic acid and 2 mM of ammonium formate. The gradient (in *v/v*%) started with 25% of B during 2 min. Then B increased linearly to 90% in 5 min. This composition was maintained for an additional 5 min and then returned to 25% of B in 0.5 min and maintained this composition for 3.5 min before the next run. The flow rate was 0.3 mL/min. The injection volume was 10 μL. 

Mass analysis was performed on an Orbitrap Elite (Thermo Scientific, Bremen, Germany) mass spectrometer with a Heated EletroSpray Ionization source (HESI-II). Data were acquired under positive and negative polarity using the following ionization parameters: spray voltages, 3.0 kV; sheath gas, 40 arbitrary units; auxiliary gas, 10 arbitrary units; heater temperature, 300 °C; capillary temperature, 350 °C; S-Lenses RF level, 64.9%. 

LC-MS analyses were performed under full scan, MRM and data-dependent modes. Full scan *m/z* range was between 100 and 1000. Data-dependent acquisition was achieved by selecting the three most intense ions under dynamic exclusion and collision-induced dissociation (CID) activation between 100 and 1500 *m/z*. MRM transitions were selected based on MS/MS spectra (CID) acquired by infusing the individual standards into the mass spectrometer at 5 μL/min. 

### 4.5. Quantitation and Profile Analysis

Quantitation was performed using profiles obtained under LC-HR-MS. AM-XIC were constructed using a mass extraction window of ±5ppm. For comparison purposes, MRM profiles were also obtained. OA, DTX-1, DTX-2 and YTX were followed in the negative polarity while AZA-1 and PTX-2 were monitored in the positive polarity.

Quantification was performed by preparing calibration curves using the areas of peaks obtained by the two procedures. Quantification limits were calculated from the standard deviations (SD) obtained after five injections of the second-lowest concentration (10*SD). The matrix effect was calculated using Equation (1).

LC-MS data analysis was performed using Xcalibur 4.1 (Thermo Scientific, Bremen, Germany). The identification of compounds was performed with Compound Discoverer 3.3 (Thermo Scientific, Bremen, Germany) by processing the data-dependent profiles with “Max ID”, “metabolomics” and “lipidomics” workflows.

ME measurements for the different analyzed fresh mussel samples were averaged, and the results are given as mean and correspondent %SRD. Averaged values of ME obtained for different batches were analyzed using one-way analysis of variance (ANOVA). Significance was accepted if the null hypothesis was rejected at *p* < 0.05. The precision of toxin concentration in extracts of contaminated samples is given as ±standard error, calculated using the extrapolation method [21].

## Figures and Tables

**Table 3:** Contents of OA and DTX-2 in contaminated samples by following a SAM and AM-XIC based quantitation, *n* = 3. Values are in ng/mL (±standard error, extrapolation method [21]).

Toxin	Sample			
	Pasteurized	Mincha	Medium	Large
OA	5.4 ± 1.1	11.6 ± 1.9	11.8 ± 1.4	14.1 ± 0.9
DTX-2	<LOQ	2.3 ± 0.7	<LOQ	3.0 ± 0.7

LOQ: Limit of Quantification.

## Supplementary Materials

The following supporting information can be downloaded at: https://www.mdpi.com/article/10.3390/md20020143/s1, Figure S1: LC-HR-MS full scan profiles and correspondent single ion chromatograms obtained after m/z extraction of values 842.5049 and 876.5104 with ±5 ppm window of a methanol standard (a) containing PTX-2 and AZA-1, and a mussel extract after alkaline hydrolysis (b) fortified with the same compounds, Figure S2: CID MS2 spectrum of OA, Figure S3: CID MS2 spectrum of DTX-1, Figure S4: CID MS2 spectrum of DTX-2, Figure S5: CID MS2 spectrum of YTX, Figure S6: CID MS2 spectrum of AZA-1, Figure S7: CID MS2 spectrum of PTX-2, Table S1: Ions, retention times, transitions and LOQ of studied toxins, Figure S8: Negative polarity average spectrum between 6.1 and 6.7 min after injection of a non-hydrolyzed extract of mussel sample, Figure S9: Positive polarity average spectrum between 6.1 and 6.7 min after injection of a non-hydrolyzed extract of mussel sample, Figure S10: Negative polarity average spectrum between 6.1 and 6.7 min after injection of a hydrolyzed extract of mussel sample, Figure S11: Positive polarity average spectrum between 6.1 and 6.7 min after injection of a hydrolyzed extract of mussel sample, Figure S12: Negative polarity average spectrum between 7.1 and 7.6 min after injection of a non-hydrolyzed extract of mussel sample, Figure S13: Positive polarity average spectrum between 7.1 and 7.6 min after injection of a non-hydrolyzed extract of mussel sample, Figure S14: Negative polarity average spectrum between 7.1 and 7.6 min after injection of a hydrolyzed extract of mussel sample, Figure S15: Positive polarity average spectrum between 7.1 and 7.6 min after injection of a hydrolyzed extract of mussel sample, Figure S16: LC-HR-MS negative polarity profiles of extracts of a CRM sample and a mussel sample, Figure S17: Negative polarity average spectrum between 6.1 and 6.7 min after injection of a non-hydrolyzed extract of CRM-Zero-Mus sample, Figure S18: LC-HR-MS profiles of extracts of Pasteurized, Mincha, Medium and Large mussels under negative polarity, Figure S19: Average negative polarity spectra of processed mussel samples between 6.1 and 6.7 min. (a) Big; (b) Medium; (c) Mincha; (d) Pasteurized, Figure S20: Negative polarity average spectrum between 6.2 and 6.4 min (OA) after injection of a non-hydrolyzed extract of mussel sample, Figure S21: Negative polarity average spectrum between 6.2 and 6.4 min (OA) after injection of an hydrolyzed extract of mussel sample, Figure S22: Negative polarity average spectrum between 6.2 and 6.4 min (OA) after injection of sample Big, Figure S23: Negative polarity average spectrum between 6.2 and 6.4 min (OA) after injection of sample Medium, Figure S24: Negative polarity average spectrum between 6.2 and 6.4 min (OA) after injection of sample Mincha, Figure S25: Negative polarity average spectrum between 6.2 and 6.4 min (OA) after injection of sample Pasteurized, Figure S26: Negative polarity average spectrum between 6.5 and 6.7 min (OA) after injection of a non-hydrolyzed extract of mussel sample, Figure S27: Negative polarity average spectrum between 6.5 and 6.7 min (OA) after injection of hydrolyzed extract of mussel sample, Figure S28: Negative polarity average spectrum between 6.5 and 6.7 min (OA) after injection of sample Big, Figure S29: Negative polarity average spectrum between 6.5 and 6.7 min (OA) after injection of sample Medium, Figure S30: Negative polarity average spectrum between 6.5 and 6.7 min (OA) after injection of sample Mincha, Figure S31: Negative polarity average spectrum between 6.5 and 6.7 min (OA) after injection of sample Pasteurized, Table S2: Matrix effects and correspondent % RSD (in-batch) on OA and DTX-2 in contaminated processed samples (n = 3).
