# Supplementary material for: Quantitation Overcoming Matrix Effects of Lipophilic Toxins in Mytilus galloprovincialis by Liquid Chromatography-Full Scan High Resolution Mass Spectrometry Analysis (LC-HR-MS)
Source: Mar Drugs. 2022 Feb 15;20(2):143. doi: 10.3390/md20020143 (PMC8880440; doi:10.3390/md20020143)
Supplement: Supplementary file 1 [file marinedrugs-20-00143-s001.zip › marinedrugs-1598004-supplementary.pdf]

Supplementary Materials

Quantitation overcoming matrix effects of lipophilic toxins in *Mytilus galloprovincialis* by liquid chromatography - full scan high resolution mass spectrometry analysis (LC-HR-MS)

Camila Q. V . Costa[1], Inês I. Afonso[1], Sandra Lage[1], Pedro Reis Costa[1,2], Adelino V. M. Canário[1] and José P. Da Silva[1],*

1 Centre of Marine Sciences (CCMAR / CIMAR LA), University of Algarve, Campus de Gambelas, 8005-139, Faro, Portugal; camilaqvdacosta@gmail.com (C.Q.V.C.); inesiafonso13@gmail.com (I.I.A.); smlage@ualg.pt (S.L.); prcosta@ipma.pt (P.R.C.); acanario@ualg.pt (A.V.M.C.); jpsilva@ualg.pt (JPDS)

2 Portuguese Institute for the Sea and Atmosphere (IPMA), Av. Brasília, 1449-006 Lisbon, Portugal

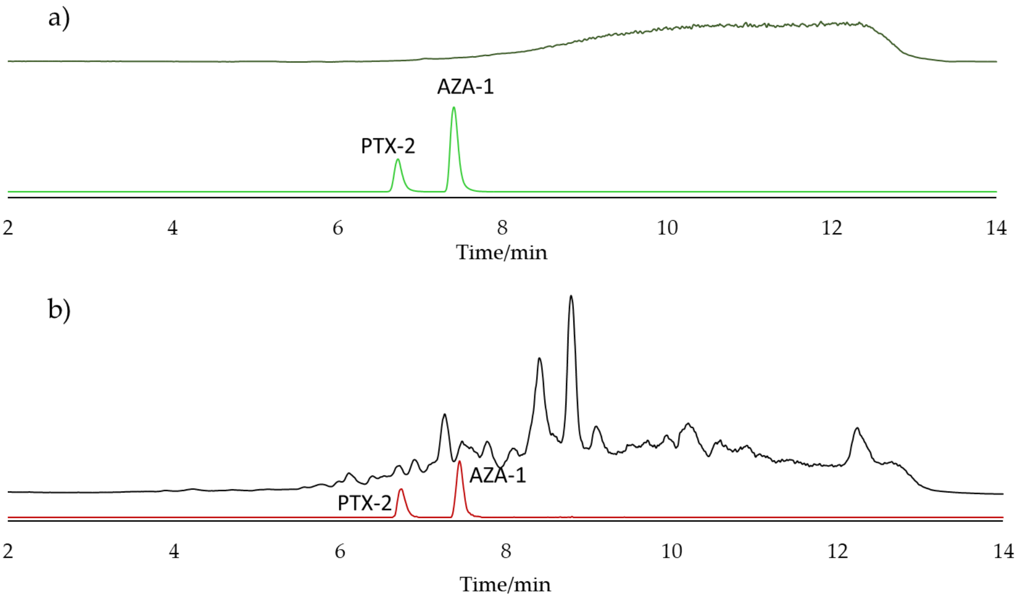

Figure S1. LC-HR-MS full scan profiles and correspondent single ion chromatograms obtained after m/z extraction of values 842.5049 and 876.5104 with ± 5 ppm window of a methanol standard (a) containing PTX-2 and AZA-1, and a mussel extract after alkaline hydrolysis (b) fortified with the same compounds.

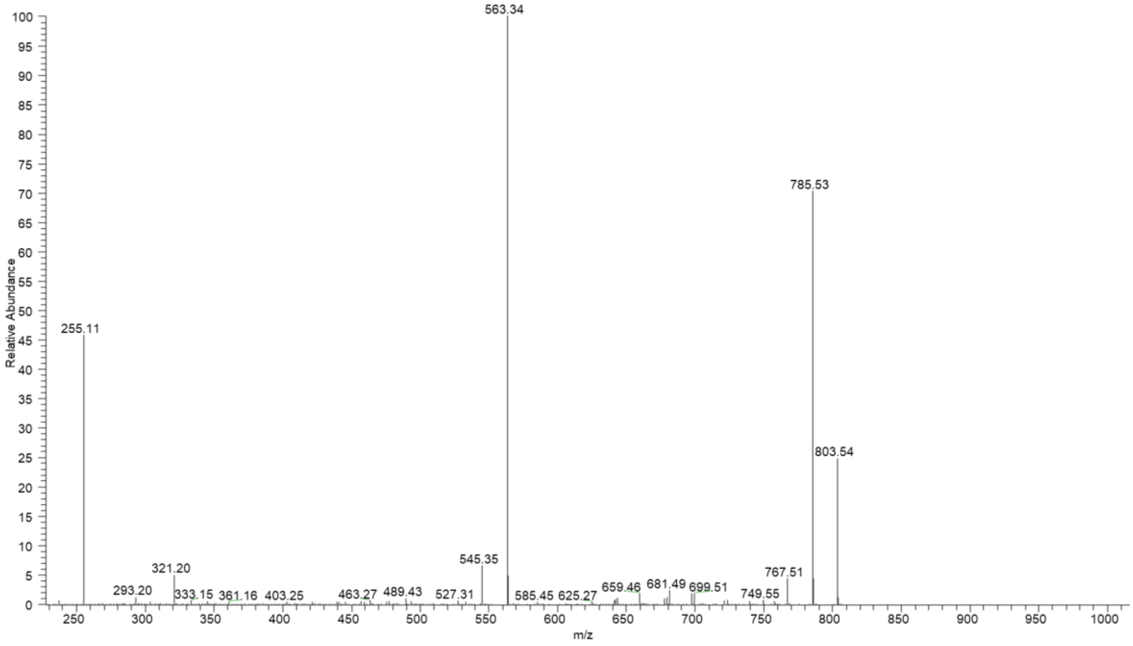

Figure S2. CID MS^2 spectrum of OA.

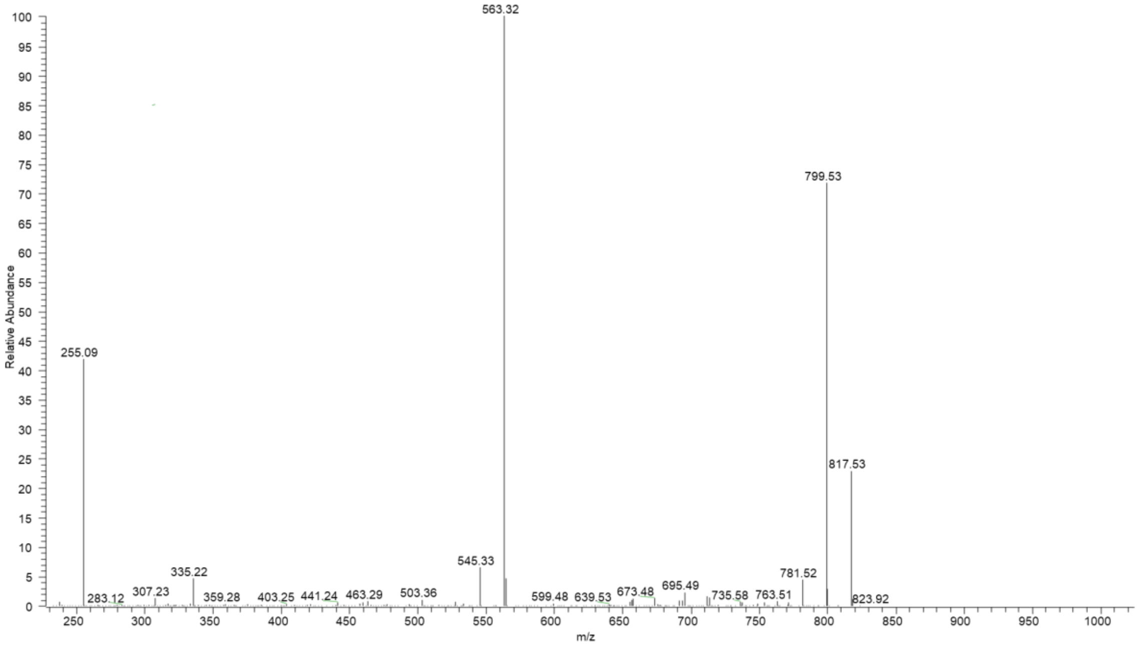

Figure S3. CID MS^2 spectrum of DTX-1.

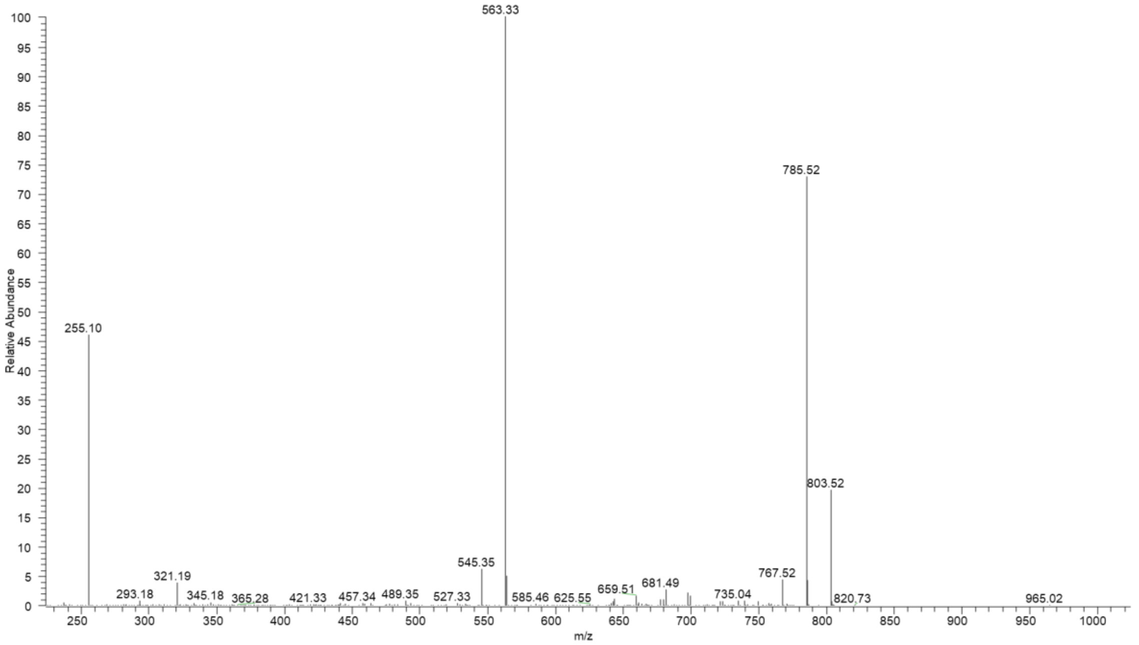

Figure S4. CID MS^2 spectrum of DTX-2.

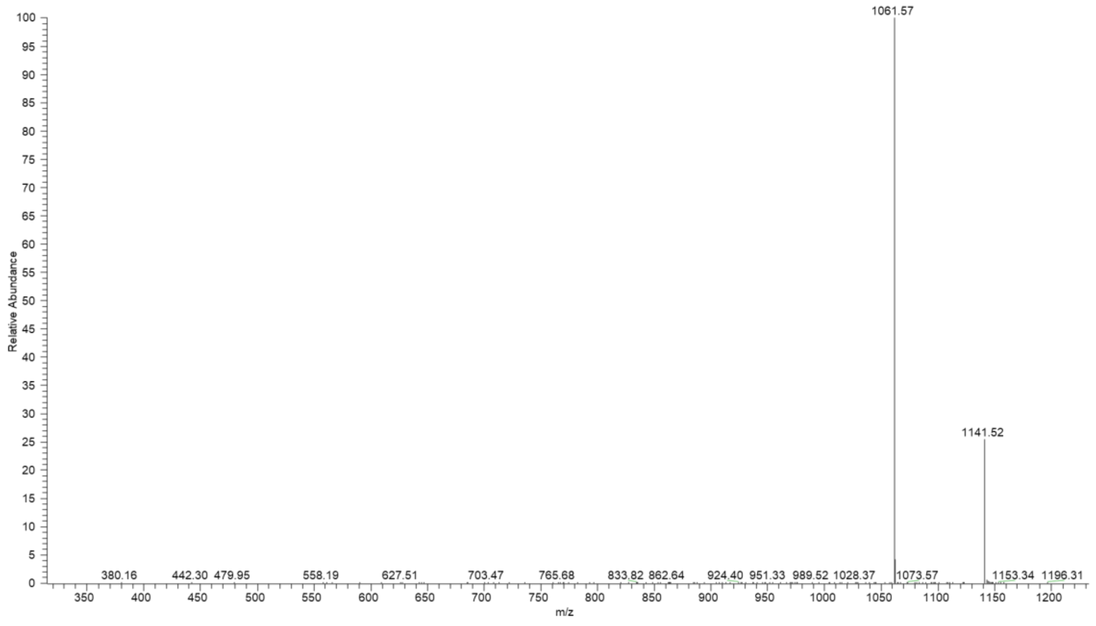

Figure S5. CID MS² spectrum of YTX.

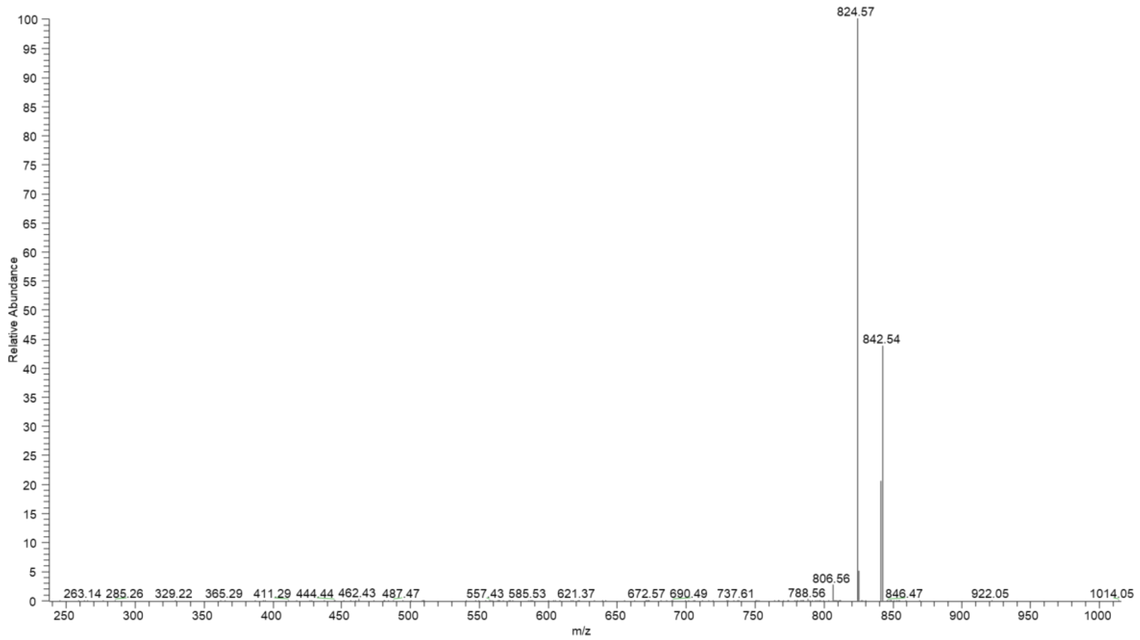

Figure S6. CID MS² spectrum of AZA-1.

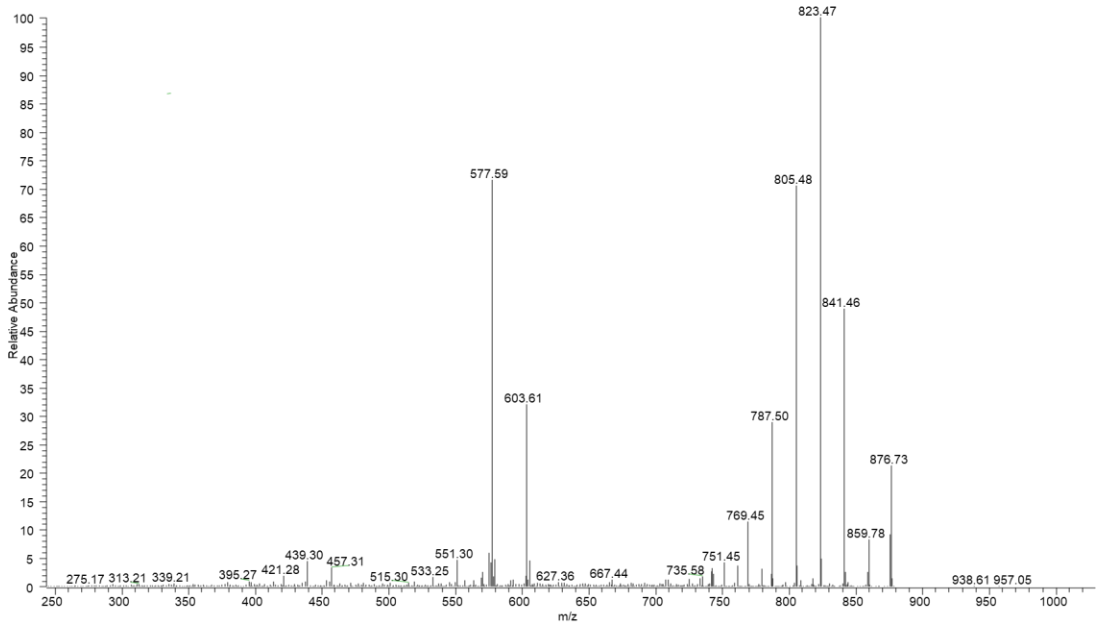

Figure S7. CID MS^2 spectrum of PTX-2.

Table S1. Ions, retention times, transitions and LOQ of studied toxins.

Toxin	Mass of ion (+/-)	RT (min)	SRM	*LOQ (ng/mL)	**LOQ (ng/mL)
OA	803.46 (-)	6.31	803 → 785; 563; 255	1.6	3.1
DTX-1	817.47 (-)	7.18	817 → 799; 563; 255	3.1	3.1
DTX-2	803.46 (-)	6.57	803 → 785; 563; 255	1.5	3.1
YTX	1141.47 (-)	6.44	1141 → 1061	2.3	1.9
AZA-1	842.50 (+)	7.45	842 → 806; 824	0.7	0.4
PTX-2	876.51 (+)	6.75	876 → 823; 805; 577	1.4	1.6

*Calculated from the standard deviation (SD) of areas obtained after 5 injections of the second lowest concentration (SD*10). Matrices were extracts of fresh mussels samples, hydrolyzed for OA, DTX-1 and DTX-2 and non-hydrolyzed for the remaining toxins. **Taken from reference Rodriguez et al., Talanta 2018, 189, 622-628.

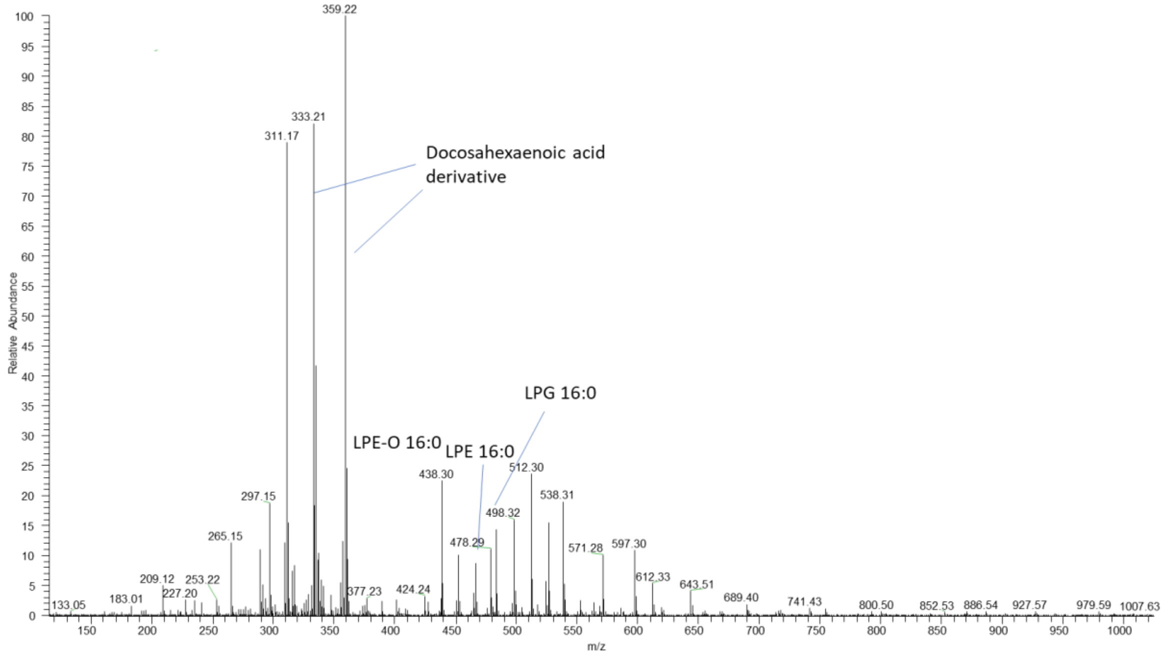

Figure S8. Negative polarity average spectrum between 6.1 and 6.7 minutes after injection of a non-hydrolyzed extract of mussel sample.

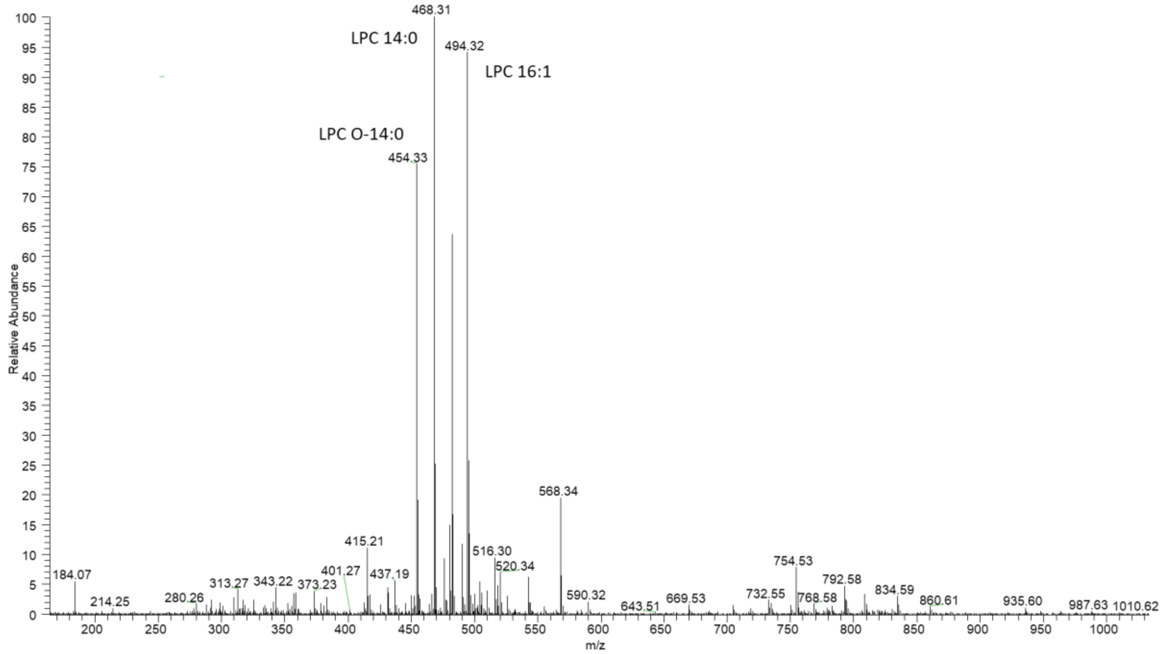

Figure S9. Positive polarity average spectrum between 6.1 and 6.7 minutes after injection of a non-hydrolyzed extract of mussel sample.

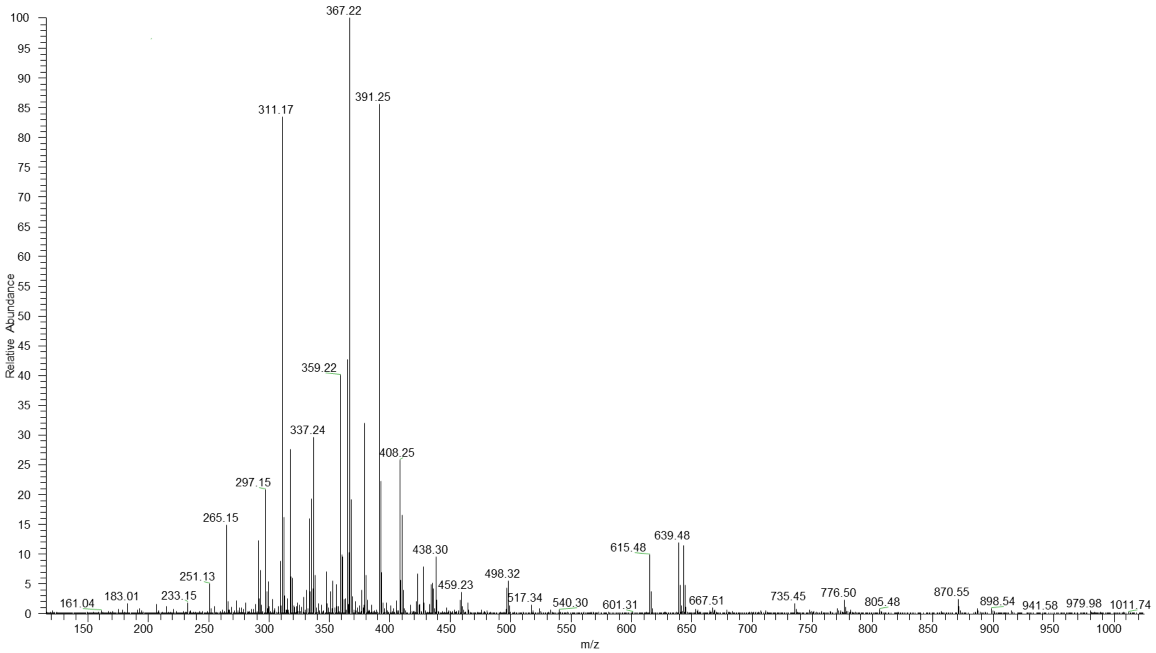

Figure S10. Negative polarity average spectrum between 6.1 and 6.7 minutes after injection of a hydrolyzed extract of mussel sample.

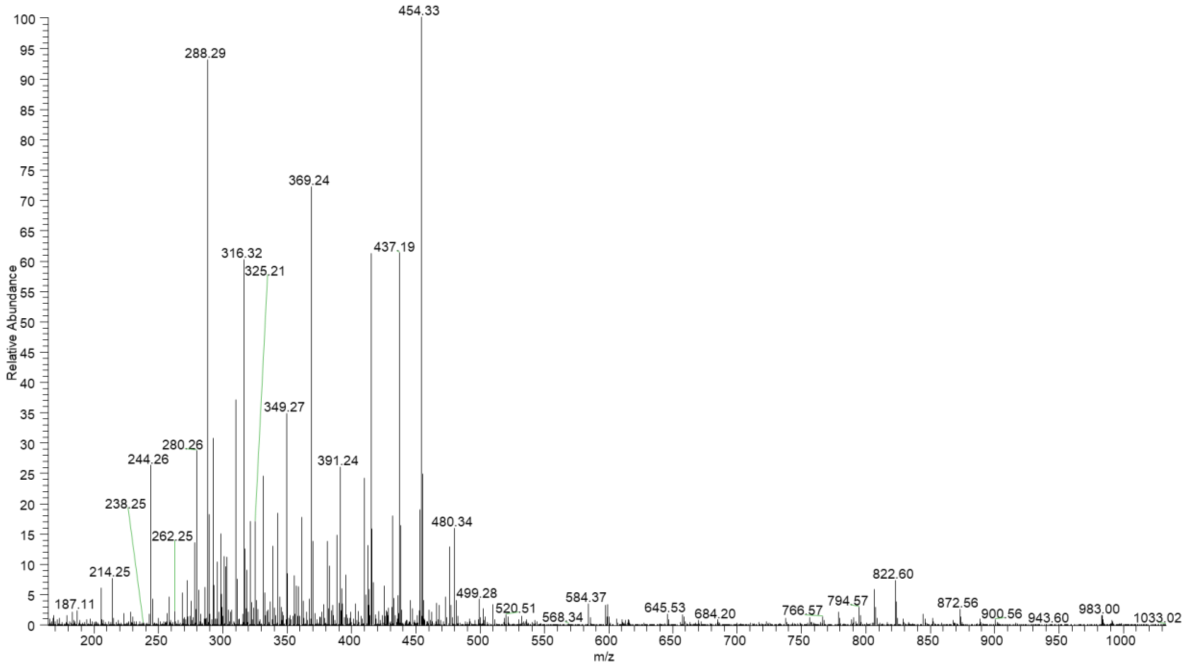

Figure S11. Positive polarity average spectrum between 6.1 and 6.7 minutes after injection of a hydrolyzed extract of mussel sample.

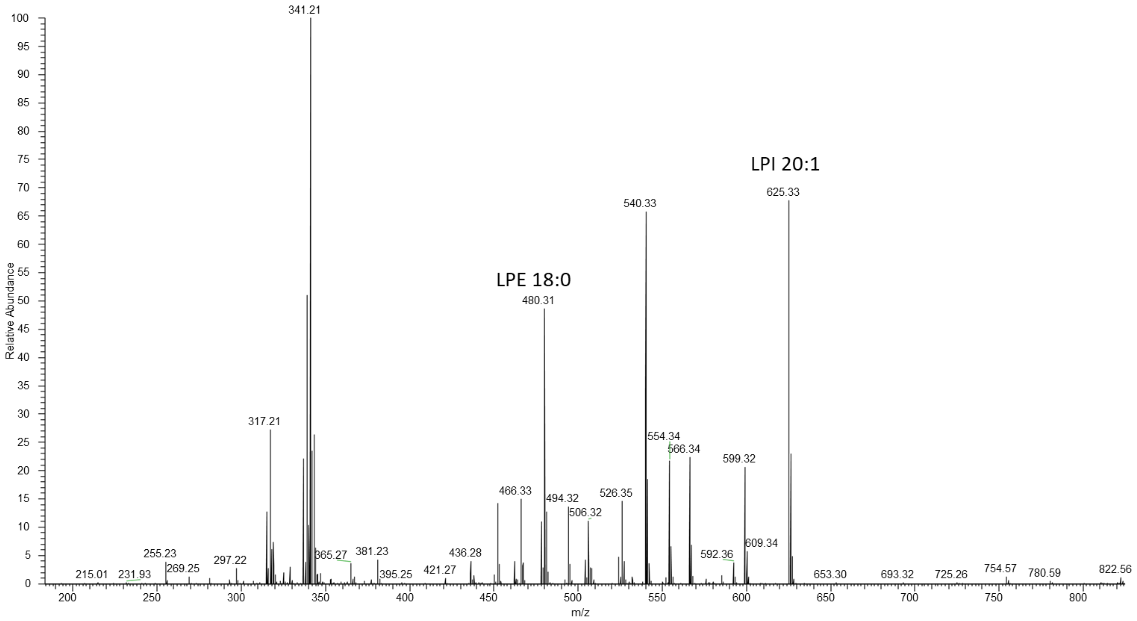

Figure S12. Negative polarity average spectrum between 7.1 and 7.6 minutes after injection of a non-hydrolyzed extract of mussel sample.

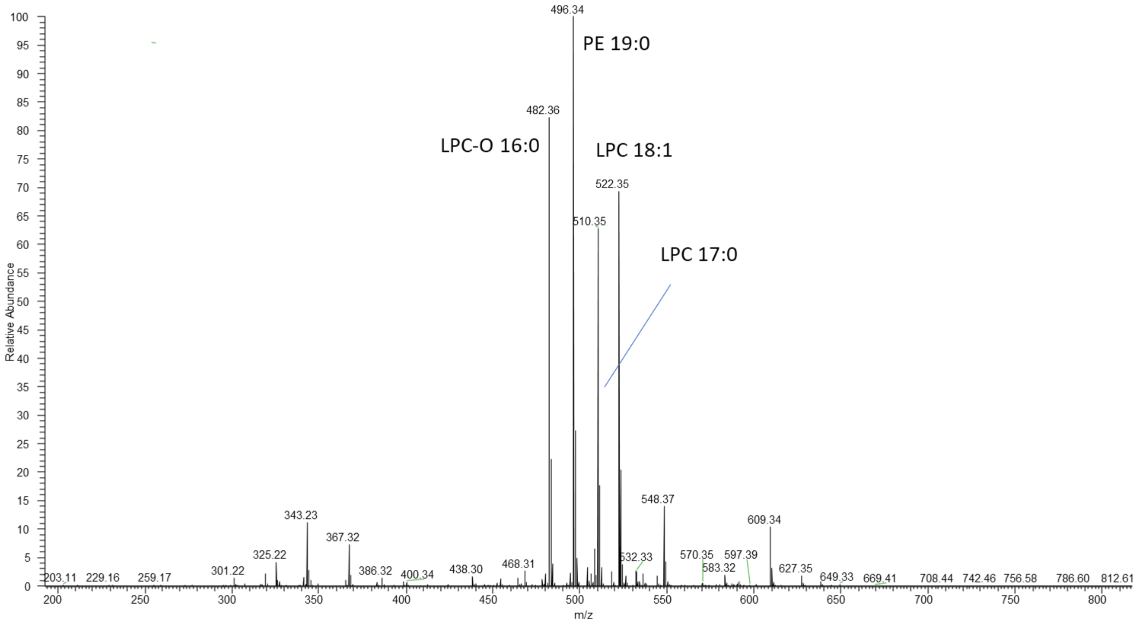

Figure S13. Positive polarity average spectrum between 7.1 and 7.6 minutes after injection of a non-hydrolyzed extract of mussel sample.

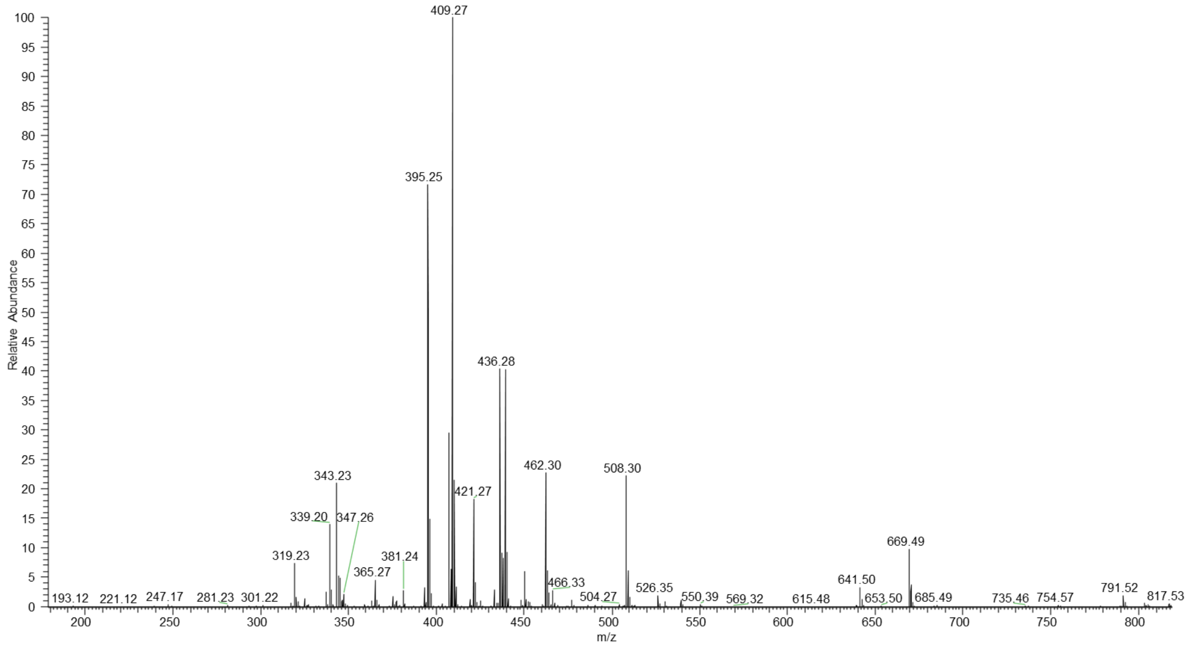

Figure S14. Negative polarity average spectrum between 7.1 and 7.6 minutes after injection of a hydrolyzed extract of mussel sample.

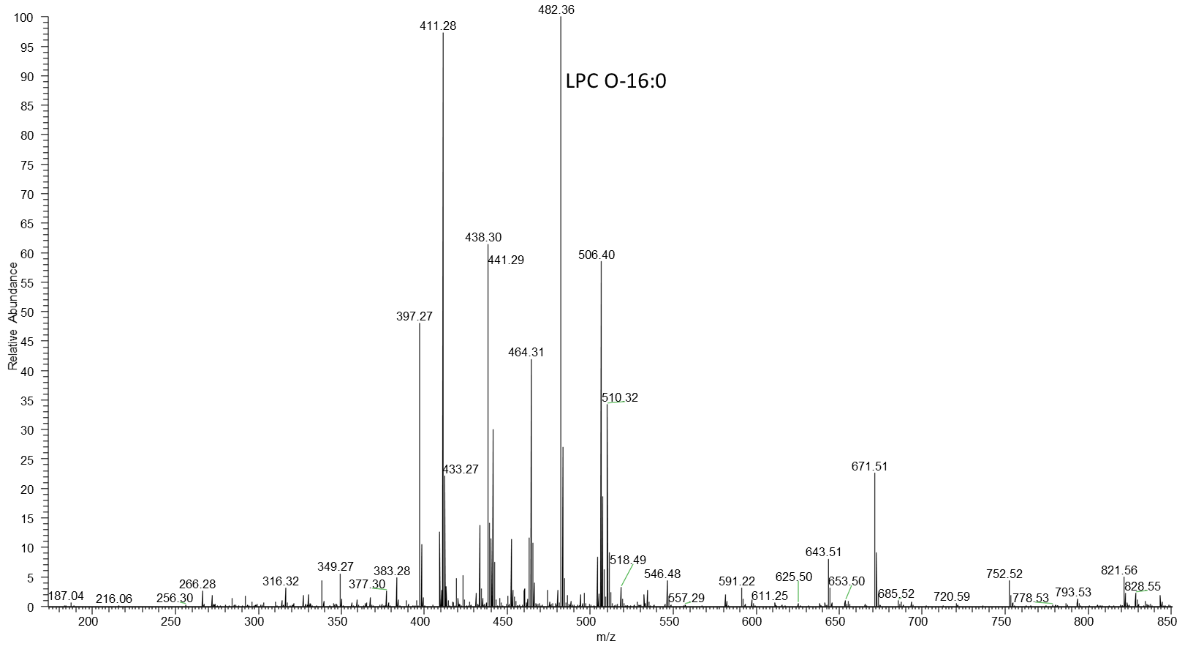

Figure S15. Positive polarity average spectrum between 7.1 and 7.6 minutes after injection of a hydrolyzed extract of mussel sample.

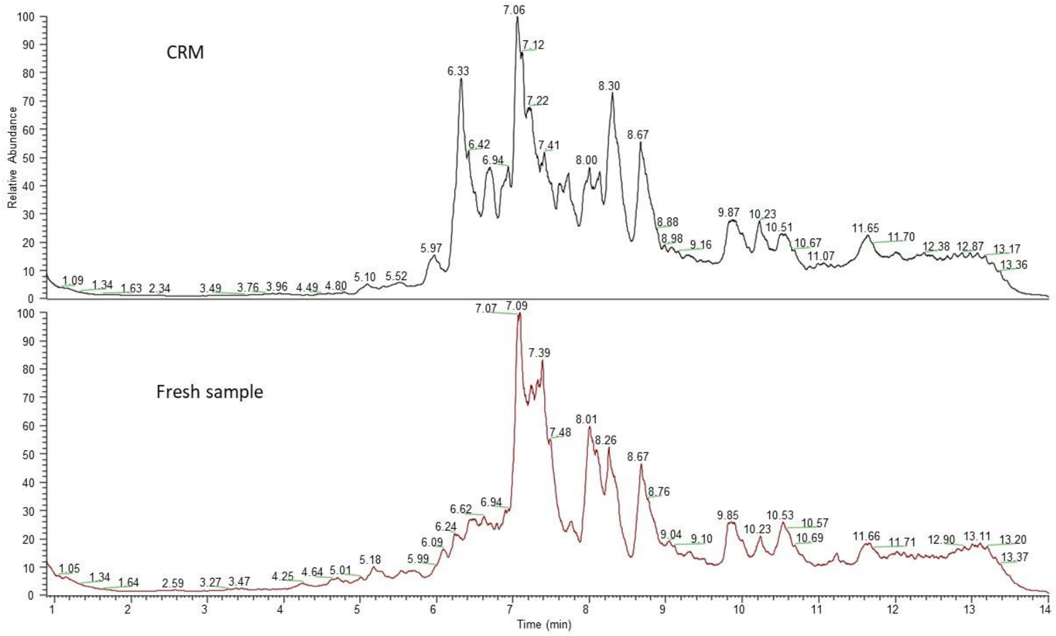

Figure S16. LC-HR-MS negative polarity profiles of extracts of a CRM sample and a mussel sample.

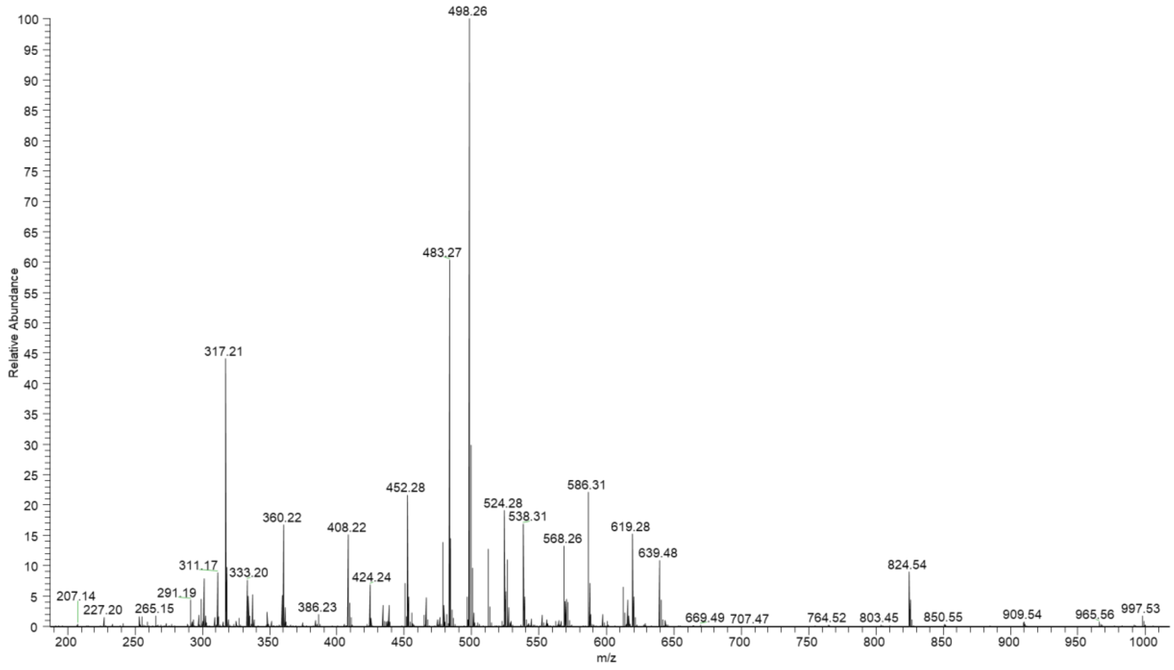

Figure S17. Negative polarity average spectrum between 6.1 and 6.7 minutes after injection of a non-hydrolyzed extract of CRM-Zero-Mus sample.

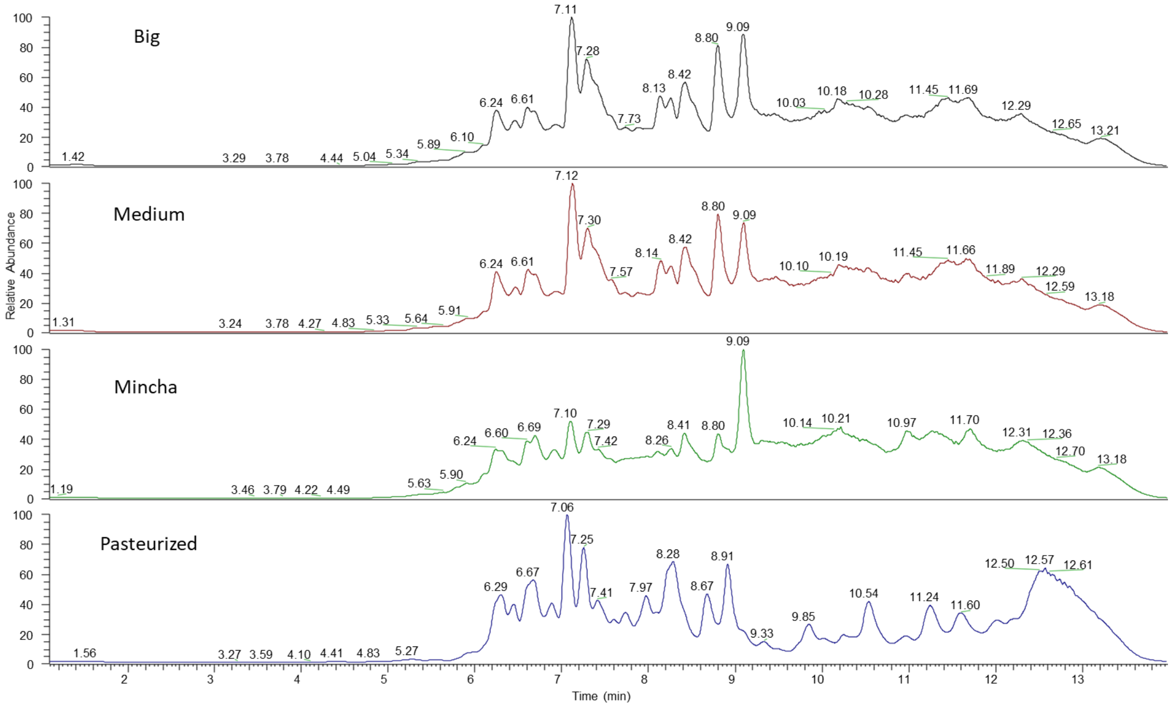

Figure S18. LC-HR-MS profiles of extracts of Pasteurized, Mincha, Medium and Large mussels under negative polarity.

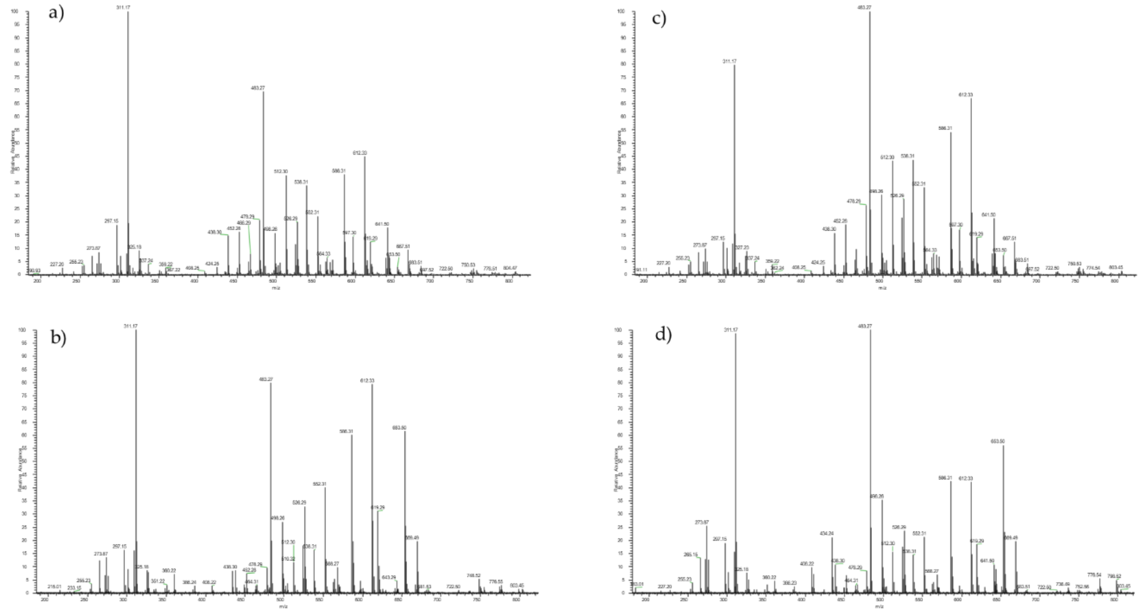

Figure S19. Average negative polarity spectra of processed mussel samples between 6.1 and 6.7 min. a) Big; b) Medium; c) Mincha; d) Pasteurized.

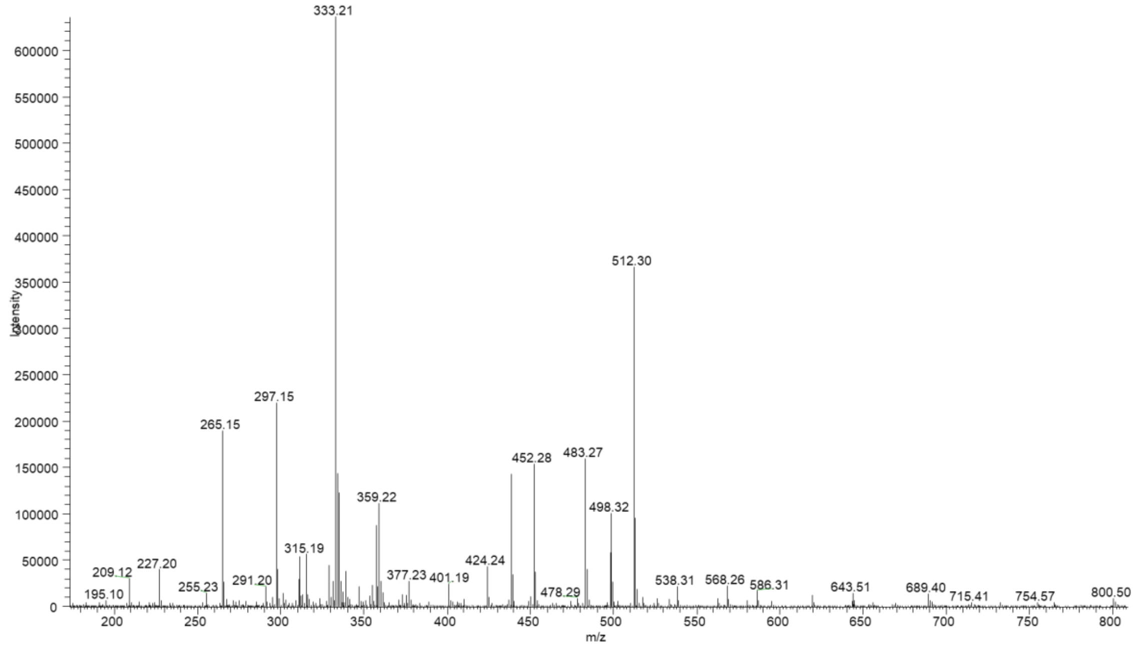

Figure S20. Negative polarity average spectrum between 6.2 and 6.4 minutes (OA) after injection of a non-hydrolyzed extract of mussel sample.

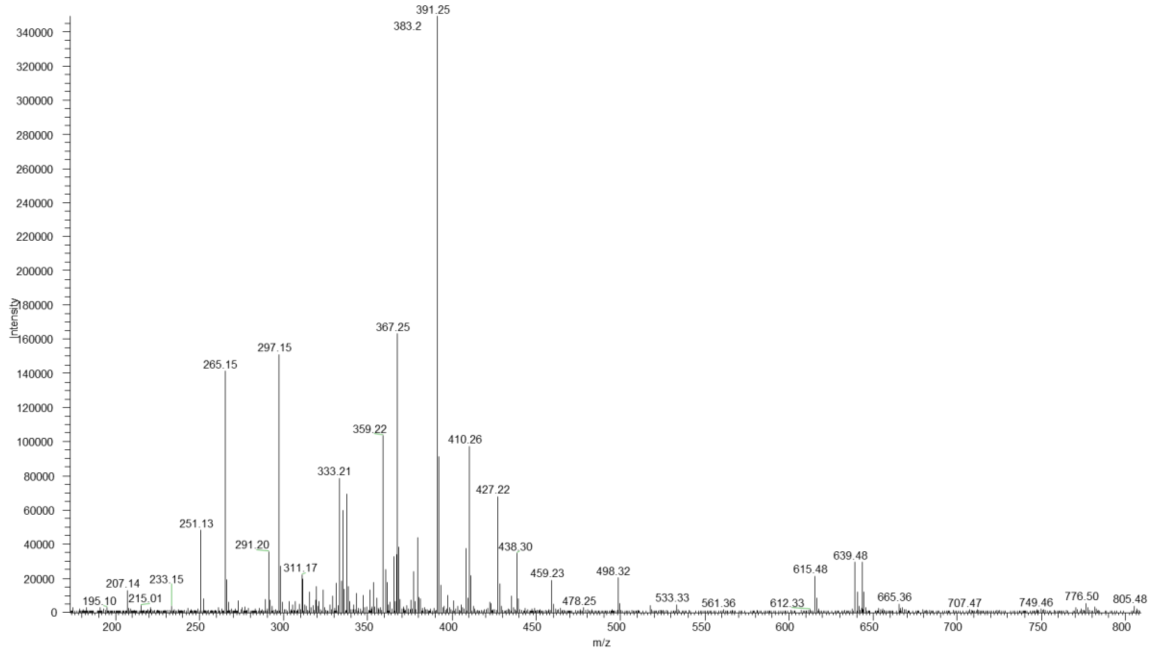

Figure S21. Negative polarity average spectrum between 6.2 and 6.4 minutes (OA) after injection of an hydrolyzed extract of mussel sample.

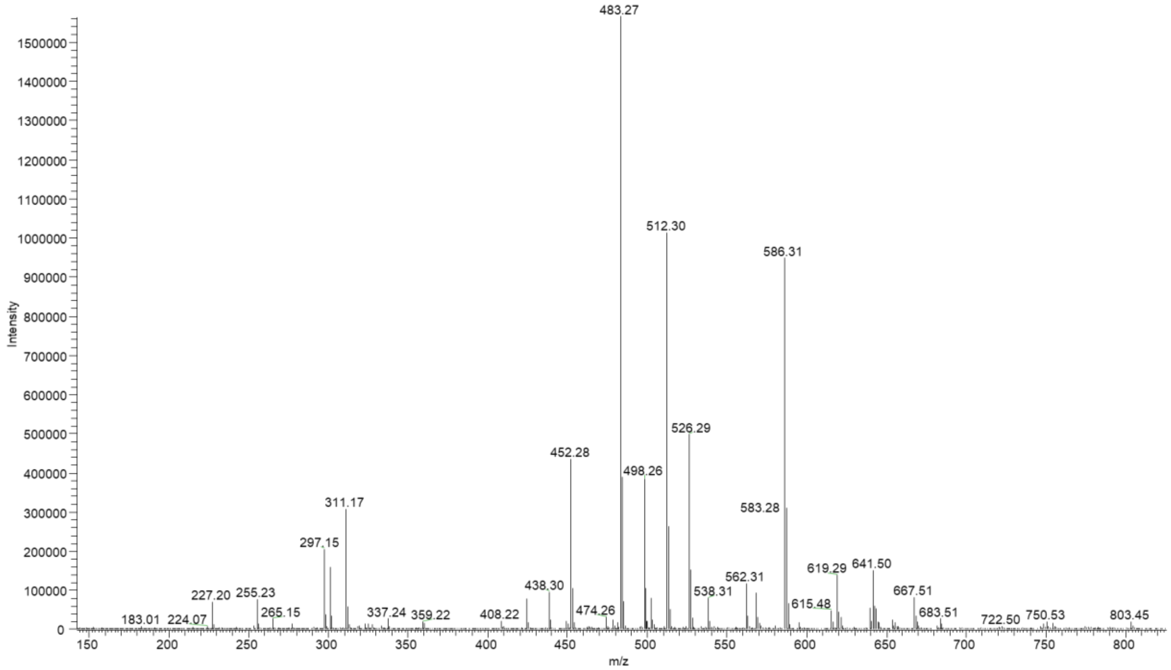

Figure S22. Negative polarity average spectrum between 6.2 and 6.4 minutes (OA) after injection of sample Big.

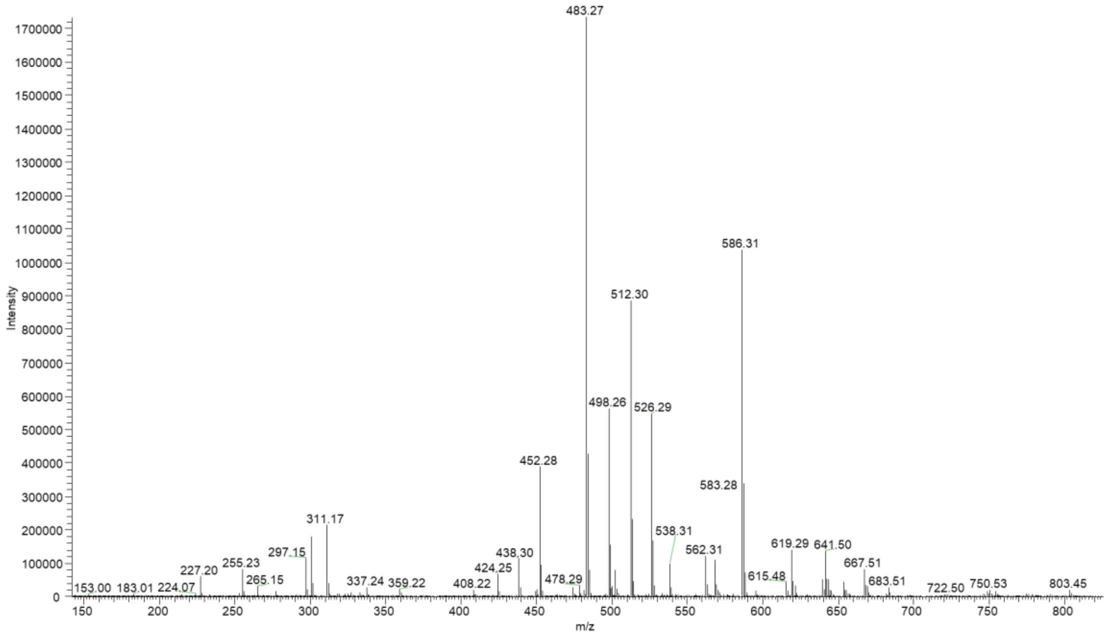

Figure S23. Negative polarity average spectrum between 6.2 and 6.4 minutes (OA) after injection of sample Medium.

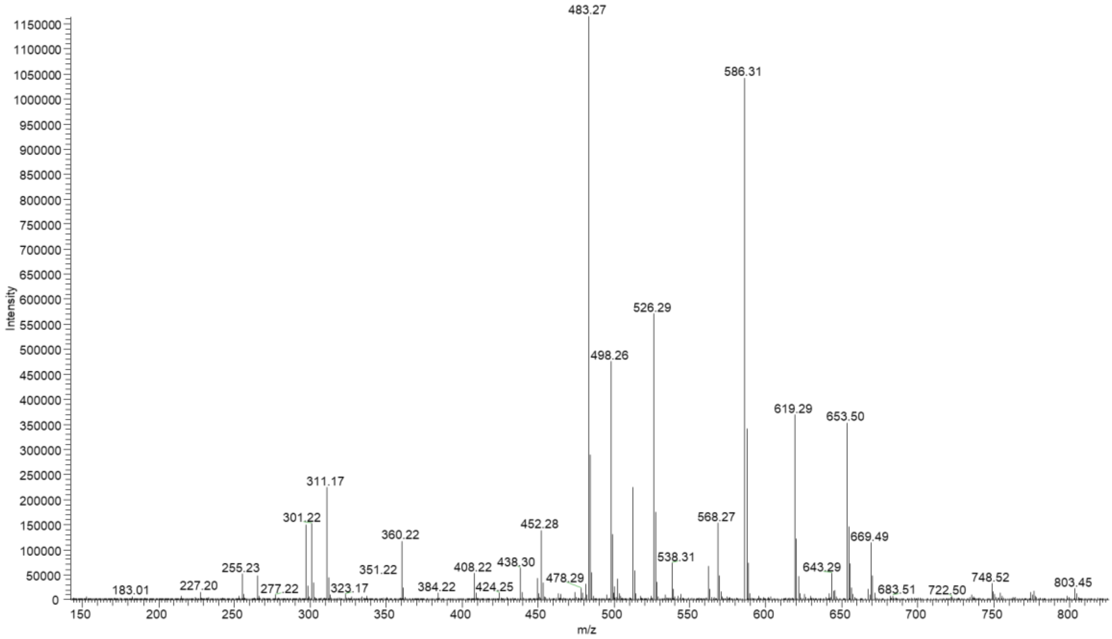

Figure S24. Negative polarity average spectrum between 6.2 and 6.4 minutes (OA) after injection of sample Mincha.

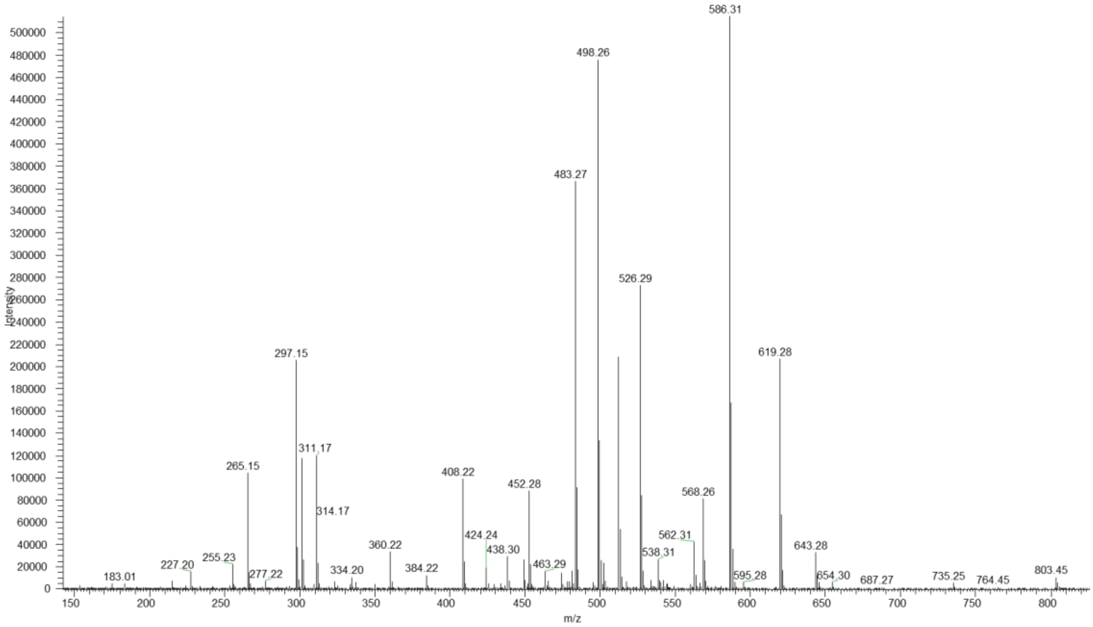

Figure S25. Negative polarity average spectrum between 6.2 and 6.4 minutes (OA) after injection of sample Pasteurized.

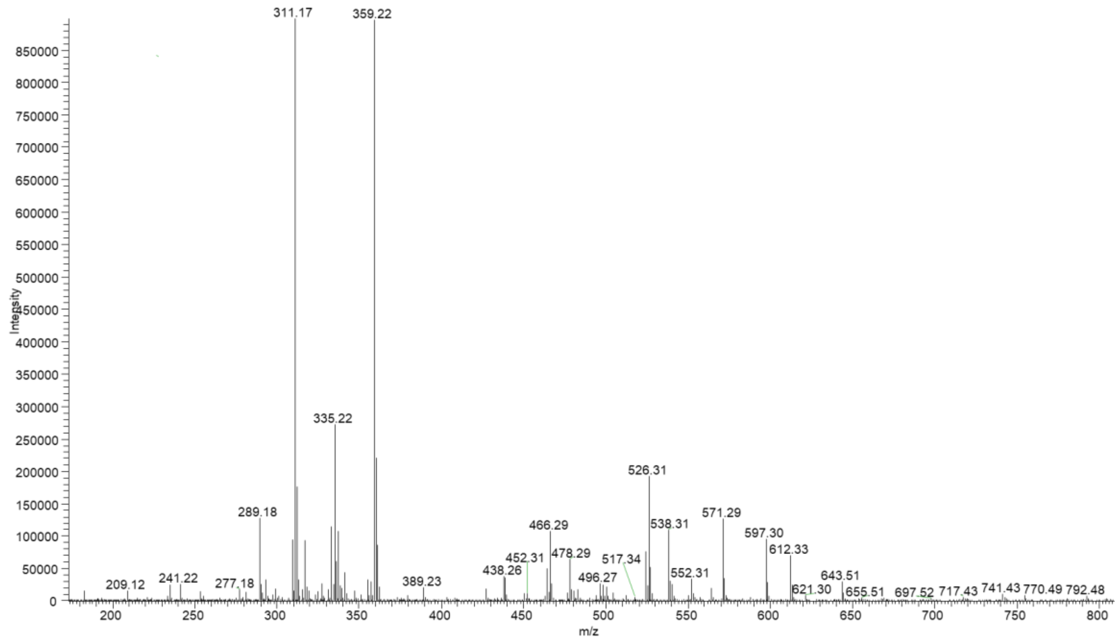

Figure S26. Negative polarity average spectrum between 6.5 and 6.7 minutes (OA) after injection of a non-hydrolyzed extract of mussel sample.

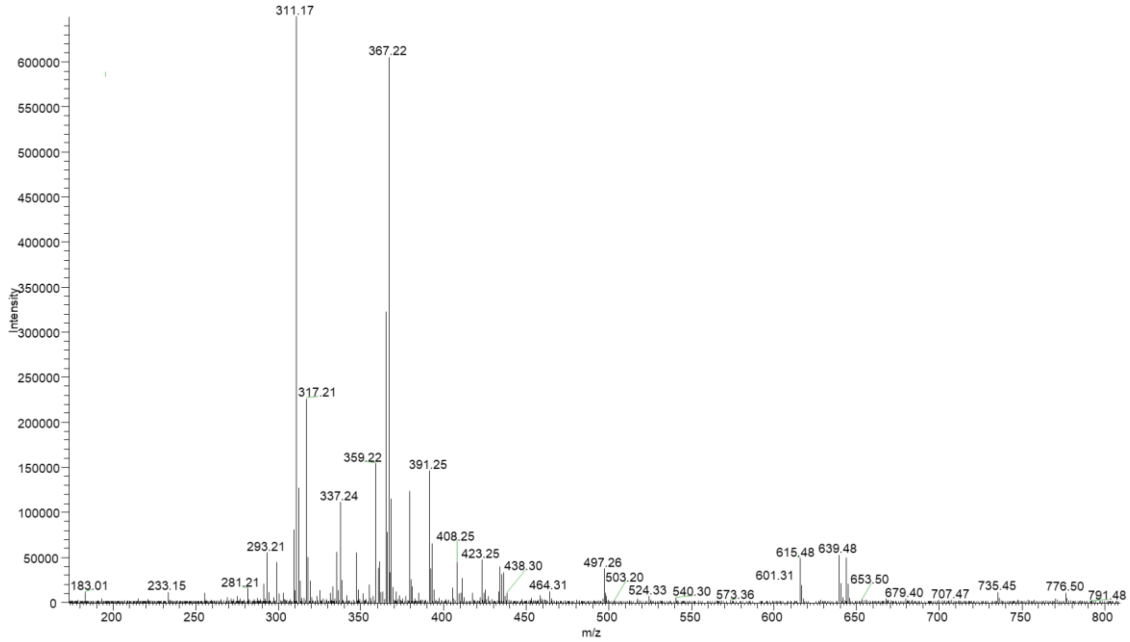

Figure S27. Negative polarity average spectrum between 6.5 and 6.7minutes (OA) after injection of hydrolyzed extract of mussel sample.

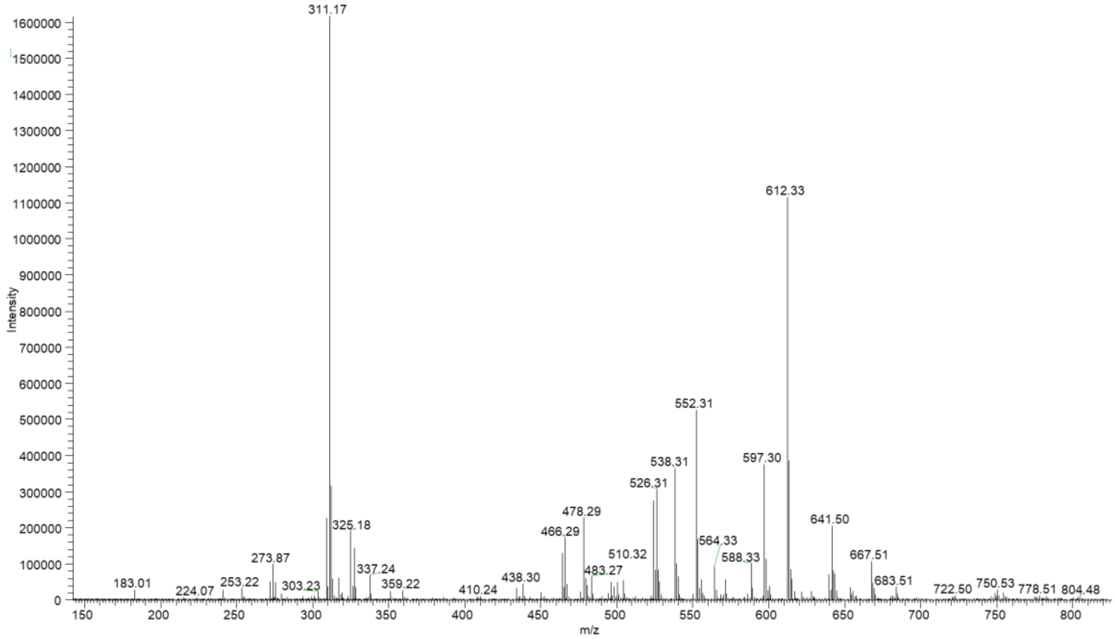

Figure S28. Negative polarity average spectrum between 6.5 and 6.7minutes (OA) after injection of sample Big.

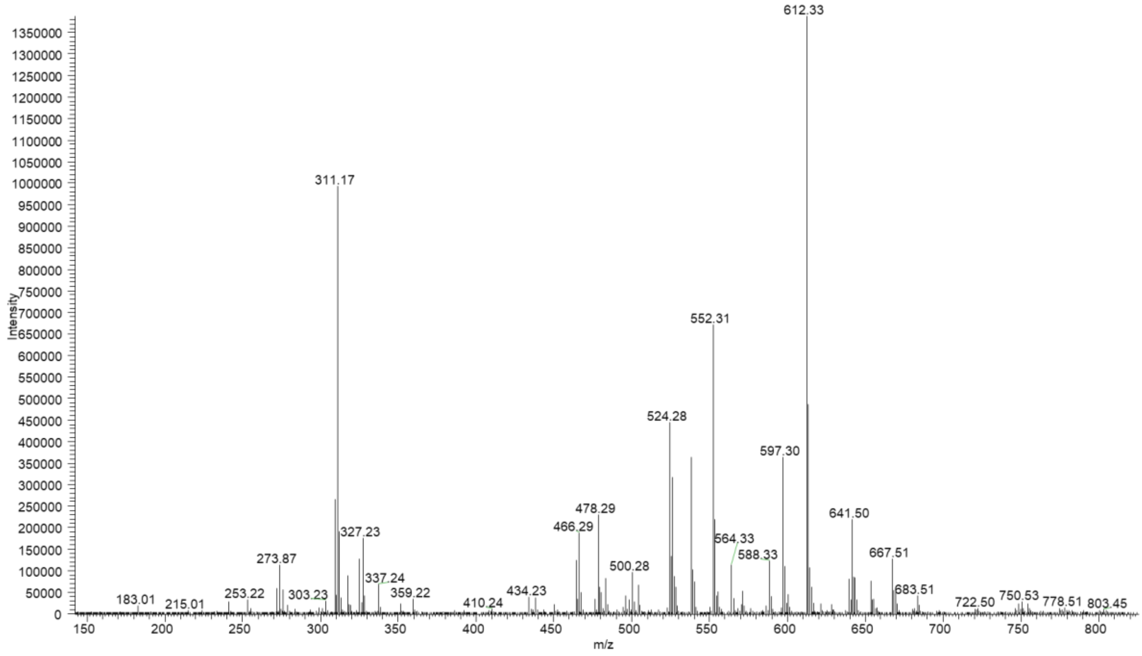

Figure S29. Negative polarity average spectrum between 6.5 and 6.7 minutes (OA) after injection of sample Medium.

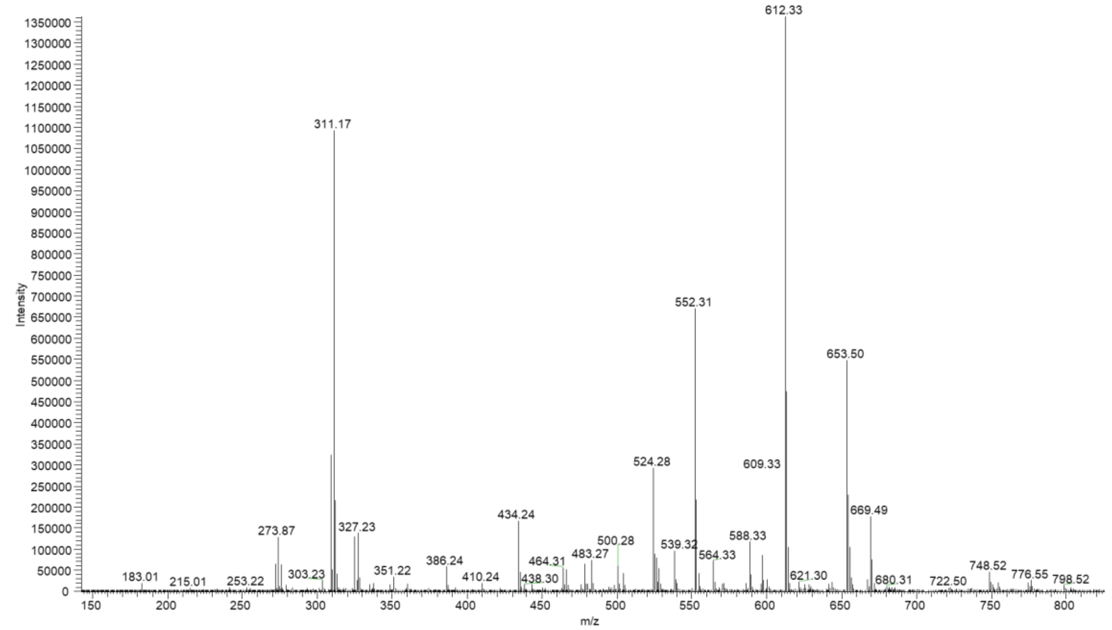

Figure S30. Negative polarity average spectrum between 6.5 and 6.7minutes (OA) after injection of sample Mincha.

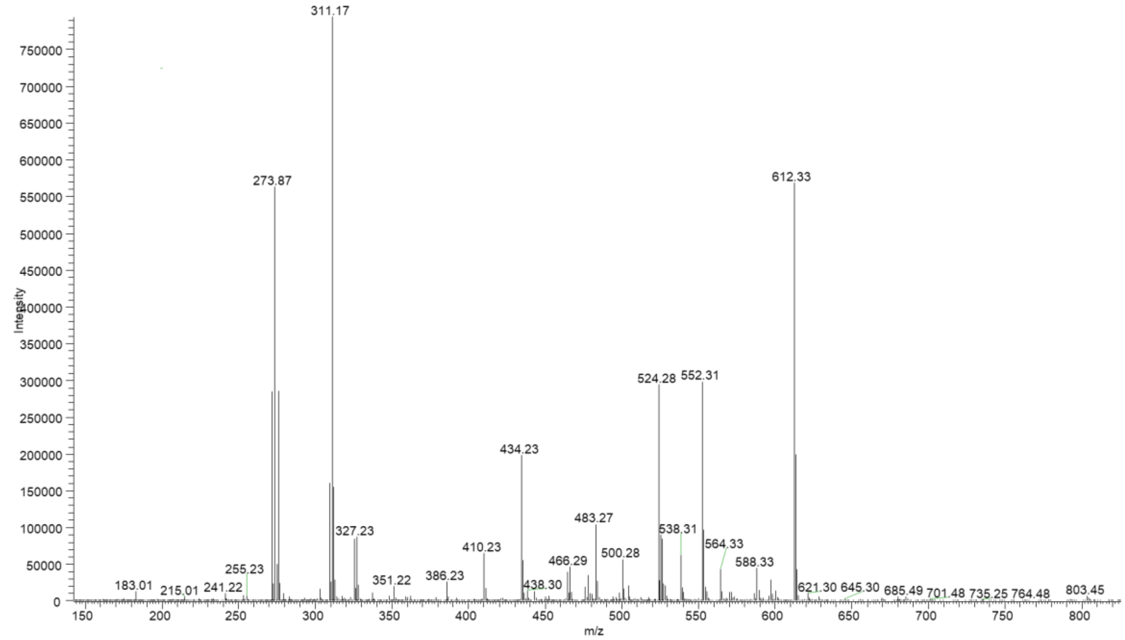

Figure S31. Negative polarity average spectrum between 6.5 and 6.7minutes (OA) after injection of sample Pasteurized.

Table S2. Matrix effects and correspondent % RSD (in-batch) on OA and DTX-2 in contaminated processed samples (n=3).

**Toxin/sample**	Big (% RSD)	Medium (% RSD)	Mincha (% RSD)	Pasteurized (% RSD)
OA	162 (5.4)	114 (4.1)	104 (7.0)	54 (7.2)
DTX-2	160 (6.2)	122 (4.8)	166 (5.4)	55 (4.9)
